# Intestinal luminal polyamines support the gut colonization of enteric bacterial pathogens by modulating flagellar motility and nitrate respiration

**DOI:** 10.1128/mbio.01786-25

**Published:** 2025-08-11

**Authors:** Tsuyoshi Miki, Shin Kurihara, Takeshi Uemura, Yuta Ami, Masahiro Ito, Takeshi Haneda, Takemitsu Furuchi, Nobuhiko Okada, Tohru Minamino, Yun-Gi Kim

**Affiliations:** 1 Department of Microbiology, School of Pharmacy, Kitasato University, Tokyo, Japan; 2 Faculty of Biology-Oriented Science and Technology, Kindai University, Wakayama, Japan; 3 Laboratory of Bio-analytical Chemistry, Faculty of Pharmaceutical Sciences, Josai University, Saitama, Japan; 4 Graduate School of Frontier Biosciences, Osaka University, Osaka, Japan; Yale University School of Medicine, New Haven, Connecticut, USA

**Keywords:** polyamines, flagellar motility, nitrate respiration, *Salmonella*, gut colonization

## Abstract

*Salmonella enterica* serovar Typhimurium (*S*Tm) is an etiological agent of common foodborne diseases and a major health concern worldwide. *S*Tm gastrointestinal infection induces intestinal inflammation, allowing *S*Tm outgrowth in the gut lumen by exploiting inflammation-induced host factors. Polyamines, including putrescine and spermidine, are crucial for both intestinal homeostasis and *S*Tm infectivity. However, our understanding of polyamines’ role in *S*Tm infection remains incomplete. This study aimed to elucidate that role. We found that defects in polyamine uptake‒dependent homeostasis altered the expression profiles of genes involved in *S*Tm flagellar motility and nitrate respiration. Spermidine supplementation restored the expression of the *nar* and *nap* operons, which encode distinct nitrate reductases, in an *S*Tm mutant with impaired polyamine homeostasis. Thus, the polyamine homeostasis-defective *S*Tm mutant exhibited reduced nitrate respiration, which spermidine supplementation rescued. In contrast, both putrescine and spermidine partially reversed the impaired expression of flagellin in this mutant. Additionally, the mutant exhibited a higher proportion of nonmotile cells compared with the wild-type strain. Finally, we demonstrated that the reduced gut colonization of this mutant was due to decreased nitrate respiration and flagellar motility. Moreover, polyamine supplementation enhanced the luminal growth of *S*Tm and a pathobiont, *Escherichia coli*. Our findings reveal that intestinal luminal polyamines support the growth of enteric bacterial pathogens in the intestinal tract.

**IMPORTANCE:**

Microbiota-derived metabolites play crucial roles in gastrointestinal infections caused by enteric pathogens. One notable example is short-chain fatty acids, such as acetate, propionate, and butyrate, which are beneficial for health and protective against infection. This study highlights that the gut microbiota‒derived polyamine spermidine drives luminal growth of enteric bacterial pathogens. The findings suggest that higher luminal levels of polyamines may be a risk factor for enteric infections. Therefore, regulating luminal polyamines could represent a promising therapeutic intervention for gastrointestinal infections.

## INTRODUCTION

*Salmonella enterica* is the leading cause of diarrheal disease globally (1). Enteritidis and Typhimurium (*S*Tm) are two highly prevalent non-typhoidal *S. enterica* serovars. Despite adequate therapeutic treatments and improved sanitation, *S*Tm gastrointestinal infection is a leading cause of infant mortality due to diarrhea worldwide (2). In healthy individuals, *S*Tm infection is typically self-limiting, with acute diarrhea lasting 3–5 days. A hallmark of infection is intestinal inflammation. In the gut lumen, flagellar motility contributes to the initial growth of *S*Tm, accompanied by the effective induction of inflammation (3). Once in the luminal gut, *S*Tm moves toward intestinal epithelial cells (IECs) across the mucosal layer through flagellum-mediated motility and chemotaxis. The bacteria then move on the cell surface in search of permissive entry sites through which to invade (4, 5). After invading the IECs, *S*Tm triggers inflammatory responses through two type-3 secretion systems (T3SSs) that inject various effectors, perturbing host cell functions (6). In the inflamed gut lumen, *S*Tm proliferates by relying on anaerobic and microaerobic respiration, utilizing host-derived oxygen and other respiratory electron acceptors, such as tetrathionate and nitrate (7–10). Flagellar motility also promotes *S*Tm growth (11), enabling sustained gut colonization at high levels for weeks to months (12, 13).

Polyamines, which include cationic aliphatic amines like putrescine (PUT), spermidine (SPD), and spermine (SPM), are ubiquitous among biological organisms (14, 15). Their ionic characteristics allow them to interact with nucleic acids, acidic phospholipids, and certain proteins (16, 17). In both healthy and diseased states of an organism, electrostatic interactions involving polyamines influence many fundamental biological functions, such as gene regulation, stress response, and cell proliferation (18). In the gut lumen, PUT and SPD are produced mainly by microbiota (19, 20). These polyamines are then imported into IECs and metabolized to SPD and SPM, influencing host physiological processes. Previous studies showed that the gut microbiota‒dependent production of polyamines is an intricate process that relies on numerous commensal bacteria (21–30), contributing to healthy and diseased states (31–35).

Previous research revealed polyamines’ involvement in the pathogenesis of various bacterial pathogens, including *Salmonella* spp. (36–44). In the context of *Salmonella* pathogenesis, a multifaceted role of polyamines has been revealed by accumulating evidence. Polyamines act as a signal, activating the expression of the virulence-associated T3SS genes (38), whereas contradictory results have been reported with regard to T3SS expression; namely, SPD facilitates the assembly of T3SS machinery (44). Furthermore, SPD participates in bacterial adherence and motility by controlling the expression of adhesins and FliA, a sigma factor for flagellar genes (42). In addition, SPD contributes to stress responses by protecting the pathogen from oxidative and nitrosative insults (40, 43). However, the precise mechanisms underlying polyamine homeostasis-dependent *S*Tm pathogenesis are not fully understood. This study investigated the role of intestinal luminal polyamines in *Salmonella* gastrointestinal infection. We found that polyamine uptake influences *S*Tm gene expression profiles, contributing to its growth in the intestinal tract. Specifically, polyamine homeostasis in *S*Tm was shown to be involved in the expression of genes related to flagellar motility and nitrate respiration. Therefore, an *S*Tm mutant strain impaired in synthesis and uptake exhibited reduced flagellar motility and nitrate respiration. Finally, we demonstrated that the uptake of luminal polyamines is necessary for *S*Tm gut colonization by activating flagellar motility and nitrate respiration. Our findings also raise the possibility that the growth supported by luminal polyamines may be generalized to other Enterobacteriaceae.

## RESULTS

### Uptake-dependent polyamine homeostasis activates genes for flagellar motility, carbohydrate metabolism, chemotaxis, and nitrate respiration

**Fig 1:**
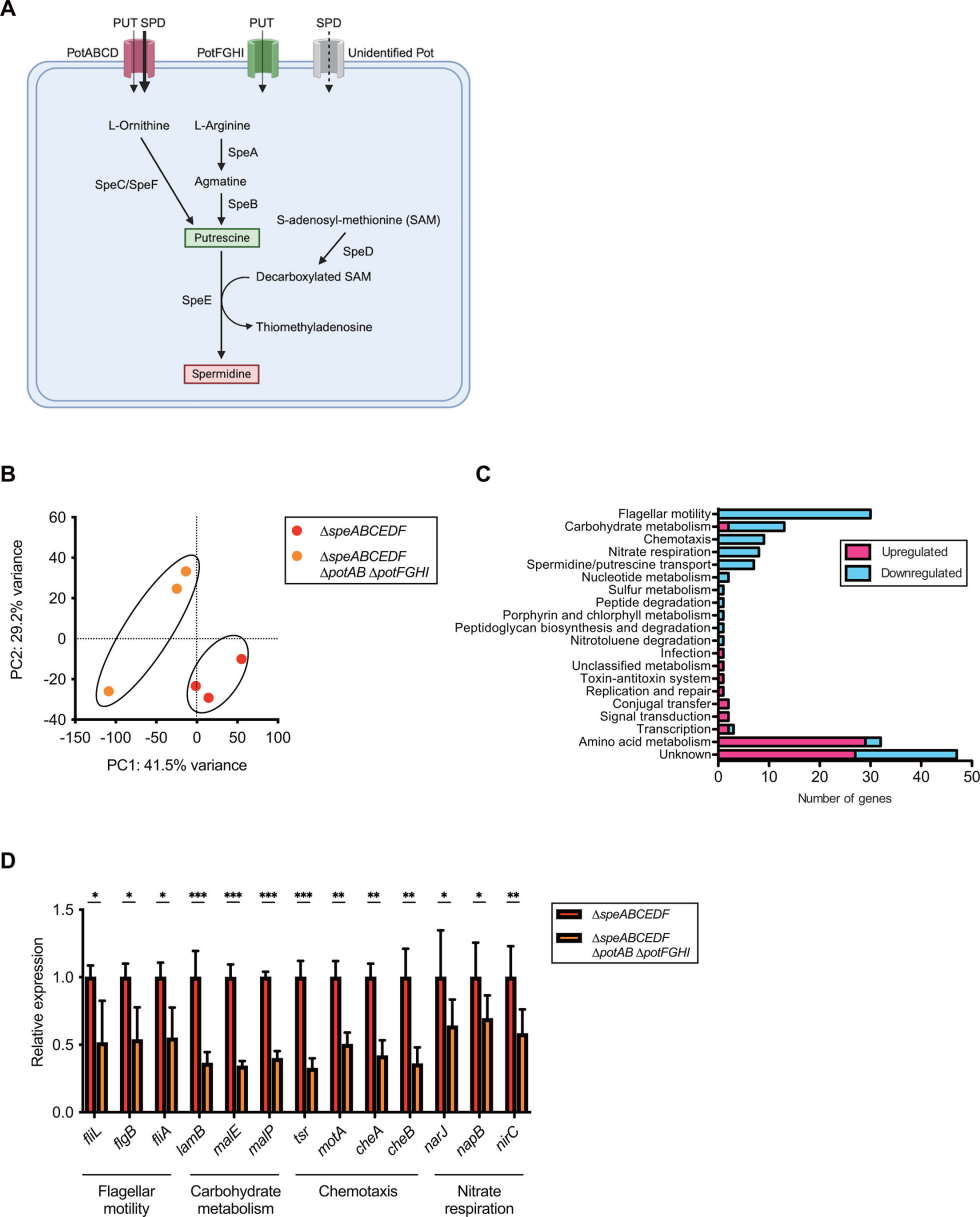
Transcriptome analyses reveal polyamine homeostasis‒dependent genes involved in flagellar motility, carbohydrate metabolism, chemotaxis, and nitrate respiration. (**A**) Representative putrescine (PUT)/spermidine (SPD) synthesis and uptake of *S*Tm. A series of Spe proteins are involved in polyamine synthesis, and two distinct transporters, composed of PotABCD and PotFGHI, predominantly contribute to polyamine uptake into the cytoplasm. Notably, *S*Tm possesses an as-yet unidentified Pot transporter that can import SPD but to a lesser degree than PotABCD (44). (**B**) Principal-component analysis (PCA) of the RNA-seq data of the ∆*speABCED* and ∆*speABCEDF* ∆*potAB* ∆*potFGHI* strains, which were grown in LB medium to mid-log phase. (**C**) Significantly differentially expressed genes (*P*_adj_ <0.01) in ∆*speABCEDF* versus ∆*speABCEDF* ∆*potAB* ∆*potFGHI* were classified according to the cluster of orthologous groups (COG) of genes. (**D**) RT-qPCR analysis of the selected genes, where their expressions are dependent on the polyamine homeostasis in *S*Tm. Data points refer to the means, where *n* is at least 3, and error bars indicate standard deviations. Unpaired Student’s *t*-test to compare two groups. *P* > 0.05 not significant (ns), *P* < 0.05 (*), *P* < 0.01 (**), *P* < 0.001 (***), *P* < 0.0001 (****).

In bacteria such as *S*Tm, a series of Spe proteins contributes to the production of PUT and SPD, whereas two PotABCD and PotFGHI transporters primarily import extracellular PUT and SPD into the bacterial cytoplasm (Fig. 1A). In the context of *S*Tm, unidentified Pot transporter(s) may also import SPD to a lesser extent when excess SPD is supplemented (44). Alternatively, the deprotonated form of these polyamines might diffuse into cells passively. Therefore, cytoplasmic levels of polyamines are tightly regulated, and uptake-dependent homeostasis plays a crucial role in *S*Tm pathogenesis (44). Thus, we investigated the intracellular contents of polyamines in *S*Tm wild-type (WT), ∆*speABCEDF* mutant lacking the ability to synthesize PUT and SPD, and ∆*speABCEDF* ∆*potAB* ∆*potFGHI* mutant impaired in the synthesis and transport of these polyamines. The *S*Tm strains were grown to the logarithmic growth phase in LB, and the bacterial precipitates were subjected to measurement by a high-performance liquid chromatography (HPLC) system equipped with a cation exchange column (44, 45). As expected, the intracellular contents of PUT and SPD were dramatically decreased in the ∆*speABCEDF* ∆*potAB* ∆*potFGHI* (Fig. S1). In contrast, the ∆*speABCEDF* apparently imported LB-derived (extracellular) SPD and thereby maintained SPD homeostasis. This was in line with the results from experiments measuring the contents of polyamines in LB medium, which revealed that LB medium used in this study contains 37.5 µM SPD, whereas neither PUT nor cadaverine was detected. Furthermore, the intracellular contents of cadaverine were not influenced by the transport of PUT and SPD. These results show that the ∆*speABCEDF* ∆*potAB* ∆*potFGHI*, but not the ∆*speABCEDF*, is defective in polyamine homeostasis when grown in LB medium.

Notably, polyamines in the bacterial cytoplasm participate in post-transcriptional regulation by binding to RNA (46). To clarify the polyamine uptake‒dependent regulation of gene expression, we performed RNA sequencing (RNA-seq) analysis and compared the gene expression profiles of the ∆*speABCEDF* ∆*potAB* ∆*potFGHI* mutant with those of the ∆*speABCEDF* mutant to identify polyamine-regulated gene expression. Principal component analysis of the RNA-seq data revealed two major clusters: one composed of genes from the ∆*speABCEDF* mutant and the other formed by genes from the ∆*speABCEDF* ∆*potAB* ∆*potFGHI* mutant (Fig. 1B). The transcript levels of 96 genes, including *potAB* and *potFGHI*, were downregulated in the ∆*speABCEDF* ∆*potAB* ∆*potFGHI* mutant compared with those in the ∆*speABCEDF* mutant (Table S1). Thus, the repertoire of genes downregulated in the ∆*speABCEDF* ∆*potAB* ∆*potFGHI* mutant fell into several broad functional classes, implicating uptake-dependent polyamine homeostasis in flagellar motility, carbohydrate metabolism, chemotaxis, and nitrate respiration (Fig. 1C). Selected genes that were downregulated in the ∆*speABCEDF* ∆*potAB* ∆*potFGHI* mutant were verified by RT-qPCR (Fig. 1D). In contrast, genes involved in amino acid metabolism were among the most strongly upregulated in the ∆*speABCEDF* ∆*potAB* ∆*potFGHI* mutant compared with the ∆*speABCEDF* mutant (Fig. 1C; Table S2). These results suggest that Pot transporter‒mediated, uptake-dependent polyamine homeostasis influences gene expression profiles in *S*Tm.

### Uptake of spermidine, but not putrescine, activates expression of the *narKGHJI* and *napFDAGHBC* operons

**Fig 2:**
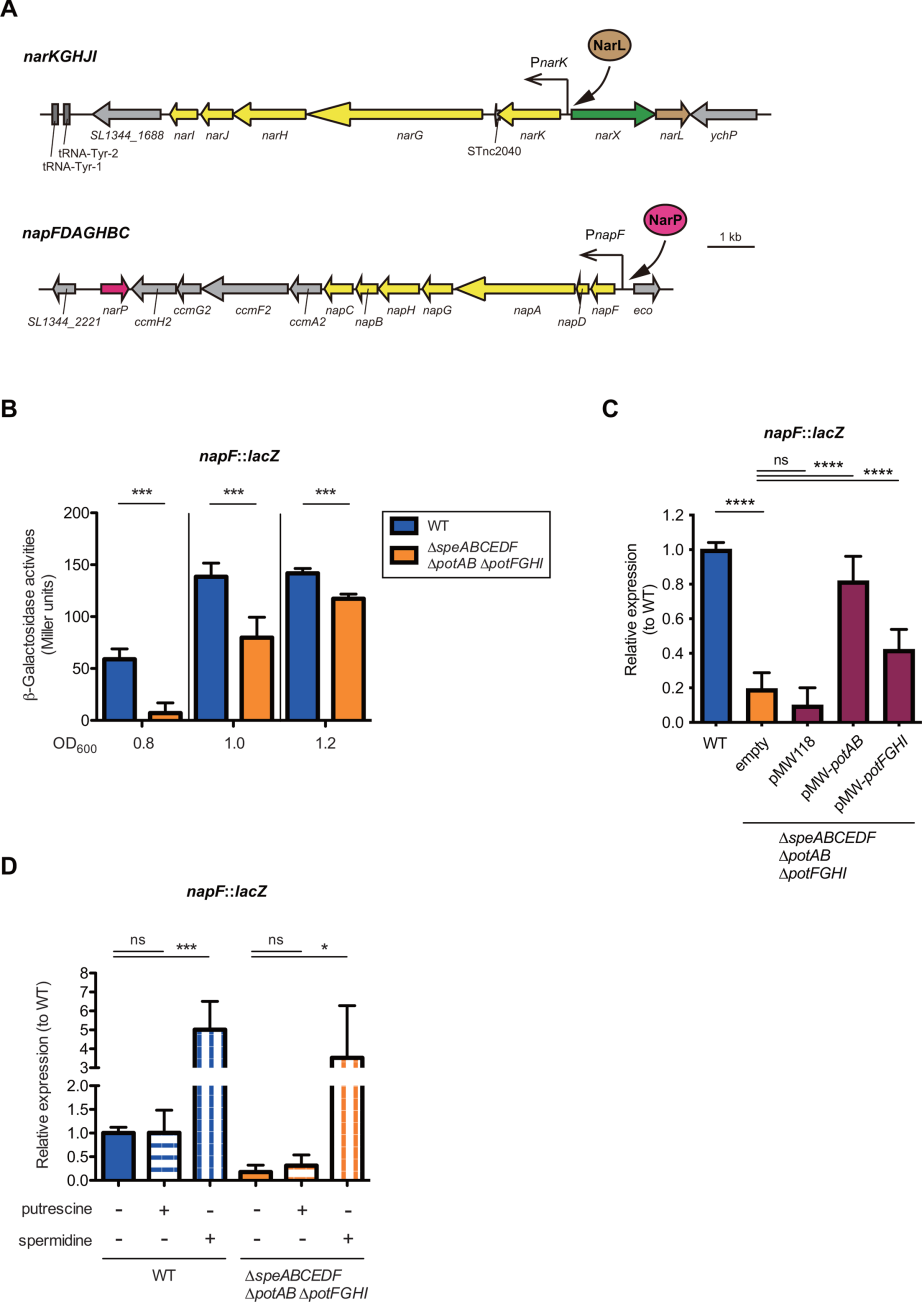
Spermidine activates expression of the nitrate reductase‒encoding operons of *narKGHJI* and *napFDAGHBC*. (**A**) Schematic of the *narKGHJI* and *napFDAGHBC* loci (yellow). (**B through D**) Transcription levels of the *napF* gene of *S*Tm grown in LB medium. Data are presented as units of β-galactosidase activity (**B**) or relative expression to WT (**C and D**). If needed, 3 mM of PUT or SPD were added to the LB medium. Bars represent mean ± SD from at least three independent experiments. Two-tailed Mann‒Whitney U tests were used to compare two groups in each panel, or a one-way ANOVA followed by Dunnett’s multiple comparisons test. *P* > 0.05 not significant (ns), *P* < 0.05 (*), *P* < 0.01 (**), *P* < 0.001 (***), *P* < 0.0001 (****).

The *narGHI* genes in the *narKGHJI* operon encode nitrate reductase A, whereas the *napABC* genes in the *napFDAGHBC* operon encode a periplasmic nitrate reductase (Fig. 2A). The RNA-seq data showed that the expression of genes in the *narKGHJI* and *napFDAGHBC* operons in the ∆*speABCEDF* ∆*potAB* ∆*potFGHI* mutant decreased, compared with the ∆*speABCEDF* mutant (Fig. S2). We next explored how polyamines activate the expression of the *napFDAGHBC* operon. To this end, a *napF::lacZ* chromosomal transcriptional fusion was constructed in both *S*Tm WT and the ∆*speABCEDF* ∆*potAB* ∆*potFGHI* mutant, and the *napF* expression levels were monitored using a β-galactosidase assay. In both strains grown in LB medium, expression depended on the growth phase, increasing during incubation (Fig. 2B). *napF* expression in the ∆*speABCEDF* ∆*potAB* ∆*potFGHI* mutant was significantly lower than that in the WT at all growth stages. These reductions were completely reversed by introducing a plasmid encoding *potAB*, whereas the introduction of an expression plasmid containing *potFGHI* only partially restored transcriptional activity (Fig. 2C). These results suggest that *napF* expression may depend on the accumulated levels of polyamines in the bacterial cytoplasm.

We next investigated the role of PUT and SPD in the expression of *napF*. Our findings showed that the addition of SPD, but not of PUT, to LB medium significantly increased *napF* expression levels in both the WT and ∆*speABCEDF* ∆*potAB* ∆*potFGHI* mutants (Fig. 2D). Furthermore, RT-qPCR analyses showed that the reduced expression of *napB* in the *napFDAGHBC* operon in the ∆*speABCEDF* ∆*potAB* ∆*potFGHI* mutant grown in nutrient minimum medium (M9) lacking polyamines was restored by supplementation with SPD but not with PUT (Fig. S3A). Similar results were obtained for *narJ* in the *narKGHJI* operon (Fig. S3B). These results indicate that SPD uptake activates both *narKGHJI* and *napFDAGHBC* operon expressions.

### Spermidine activates *napF* post-transcriptionally

**Fig 3:**
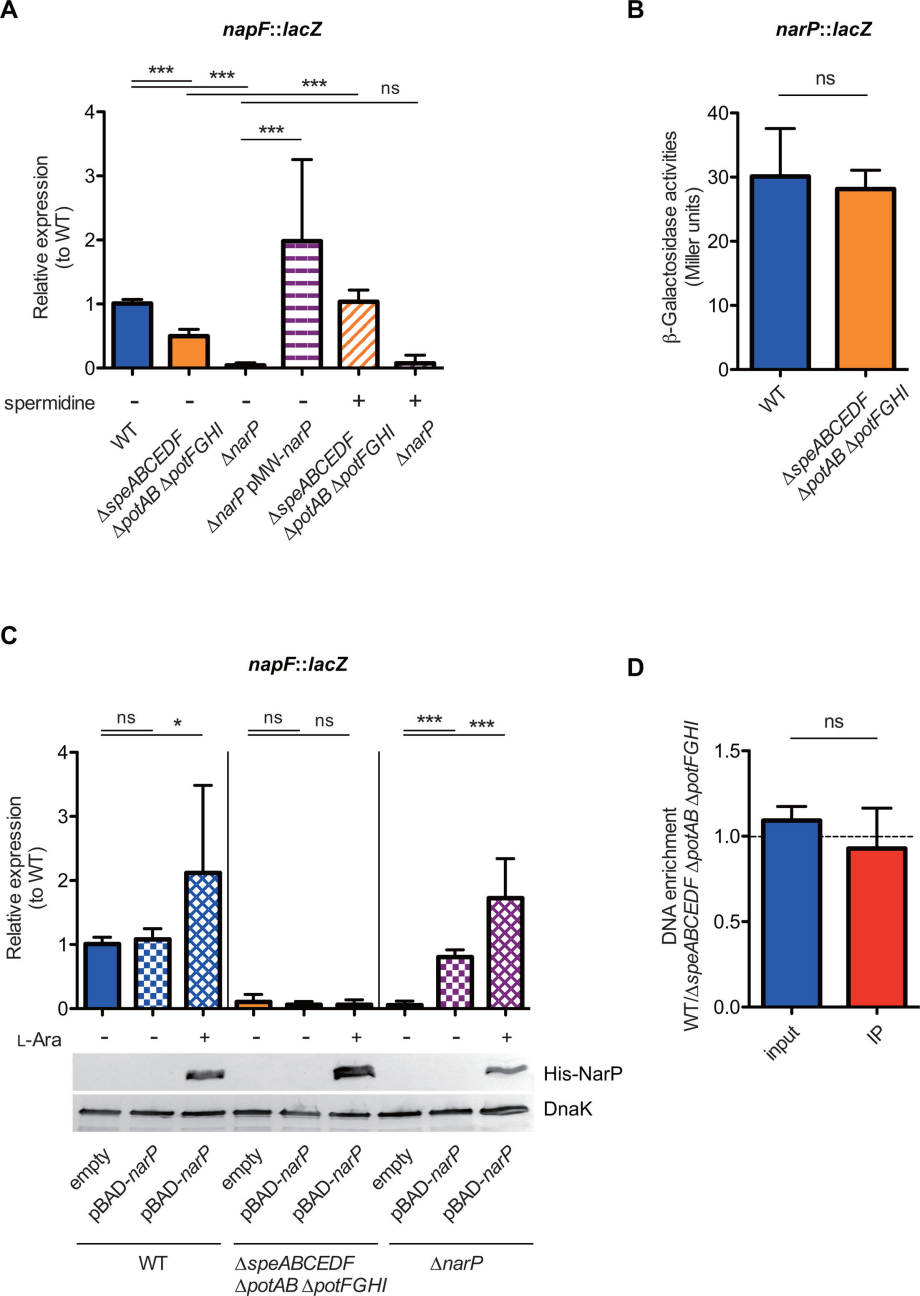
Post-transcriptional regulation of *napF* by spermidine. (**A**) Transcription levels of the *napF* gene of *S*Tm grown in LB medium are presented as relative expression to WT, determined by the β-galactosidase assay. If needed, 3 mM of SPD was added to the LB medium. (**B**) Transcription levels of the *narP* gene of *S*Tm grown in LB medium are shown as units of β-galactosidase activity. (**C**) Transcription levels of the *napF* gene of *S*Tm grown in LB medium were assessed as relative expression to WT using the β-galactosidase assay, and the expression of His-NarP and DnaK proteins was validated through western blot analysis. If needed, L-arabinose was added to the LB medium at 0.002%. (**D**) Measurement of NarP protein binding to the *napF* promoter. NarP-bound DNA was isolated via ChIP and quantified using real-time PCR. Ct values were normalized to those of the *rpoD* gene. The results are presented as ratios between values measured in WT and ∆*speABCEDF* ∆*potAB* ∆*potFGHI*, both harboring pBAD-*narP* expressing His-tagged NarP. Bars represent mean ± SD from at least three independent experiments. IP, immunoprecipitation. Two-tailed Mann‒Whitney U tests were used to compare two groups in each panel or a one-way ANOVA followed by Dunnett’s multiple comparisons test. *P* > 0.05 not significant (ns), *P* < 0.05 (*), *P* < 0.01 (**), *P* < 0.001 (***), *P* < 0.0001 (****).

The response regulator NarP positively influences *napF* expression (Fig. 2A), as demonstrated by the reduced expression levels in the ∆*narP* mutant, which were restored by introducing a plasmid encoding *narP* (Fig. 3A). In contrast, adding SPD to the growth medium had no effect on *napF* expression in the ∆*narP* mutant. Thus, SPD-mediated *napF* expression appears to depend on NarP activation.

We next investigated whether polyamine homeostasis affects *narP* expression. To this end, a β-galactosidase assay was performed using the WT and ∆*speABCEDF* ∆*potAB* ∆*potFGHI* strains harboring the *narP::lacZ* chromosomal transcriptional fusion. The transcriptional activities of *narP* in the ∆*speABCEDF* ∆*potAB* ∆*potFGHI* strains were equivalent to those in the WT (Fig. 3B), suggesting that SPD levels do not influence *narP* expression. Furthermore, although inducible expression of NarP from a plasmid increased *napF* expression in both the WT and the ∆*narP* mutant, the increased NarP did not restore the transcriptional activity of *napF* in the ∆*speABCEDF* ∆*potAB* ∆*potFGHI* strains (Fig. 3C). On the other hand, NarP binds to the promoter of *napF* at similar levels in both the WT and ∆*speABCEDF* ∆*potAB* ∆*potFGHI* strains (Fig. 3D). Collectively, these results suggest that SPD regulates *napF* expression post-transcriptionally.

### Spermidine uptake is necessary for nitrate respiration

**Fig 4:**
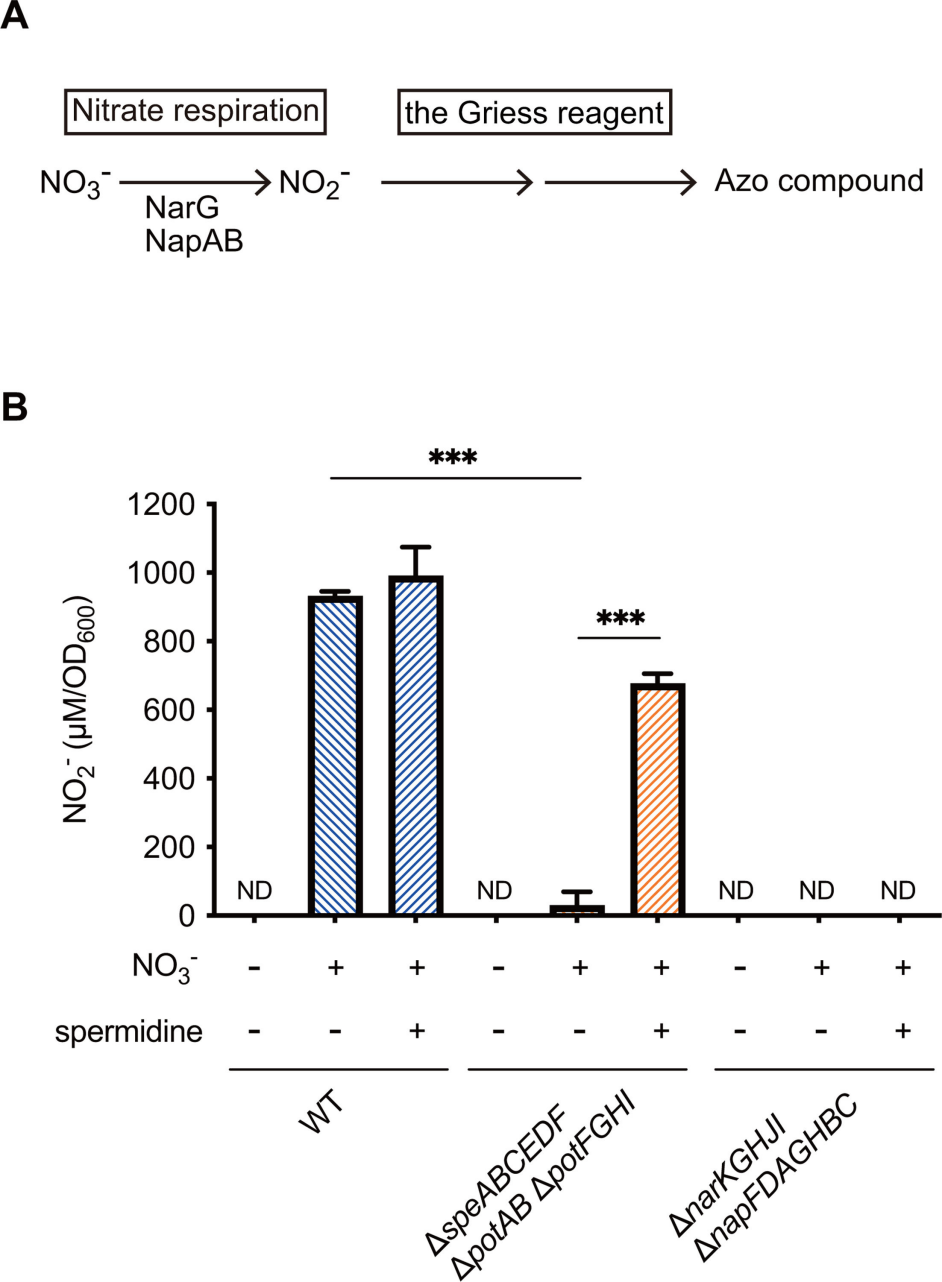
Spermidine uptake is required for the nitrate respiration of *S*tm. (**A**) Schematic of nitrate respiration and the NO_2_/NO_3_ assay based on the Griess reaction. (**B**) Activities of nitrate reductase of the indicated *S*Tm strains in the presence or absence of 40 mM sodium nitrate are assessed via the NO_2_/NO_3_ assay kit-C II (DOJINDO). If needed, 3 mM of SPD was added to the LB medium. ND, not detected. Bars represent mean ± SD from at least three independent experiments. A one-way ANOVA followed by Dunnett’s multiple comparisons test. *P* > 0.05 not significant (ns), *P* < 0.05 (*), *P* < 0.01 (**), *P* < 0.001 (***), *P* < 0.0001 (****).

We next examined whether SPD uptake influences respiratory nitrate reductase activity (47), as measured using the Griess reagent (Fig. 4A). Although the WT exhibited nitrate respiration, the ∆*narKGHJI* ∆*napFDAGHBC* mutant completely lacked respiratory nitrate reductase activity (Fig. 4B). Similarly, nitrate reductase activity was barely detectable in the ∆*speABCEDF* ∆*potAB* ∆*potFGHI* mutant. Furthermore, SPD supplementation reversed the reduction in nitrate reductase activity in the ∆*speABCEDF* ∆*potAB* ∆*potFGHI* mutant but had no effect in the ∆*narKGHJI* ∆*napFDAGHBC* mutant (Fig. 4B). These results indicate that nitrate respiration in *S*Tm depends on polyamine homeostasis, attributed to the activation of *narGHI* and *napABC* expression.

### Polyamines impact flagellum-mediated motility of *S*Tm by affecting the expression of flagellar genes

**Fig 5:**
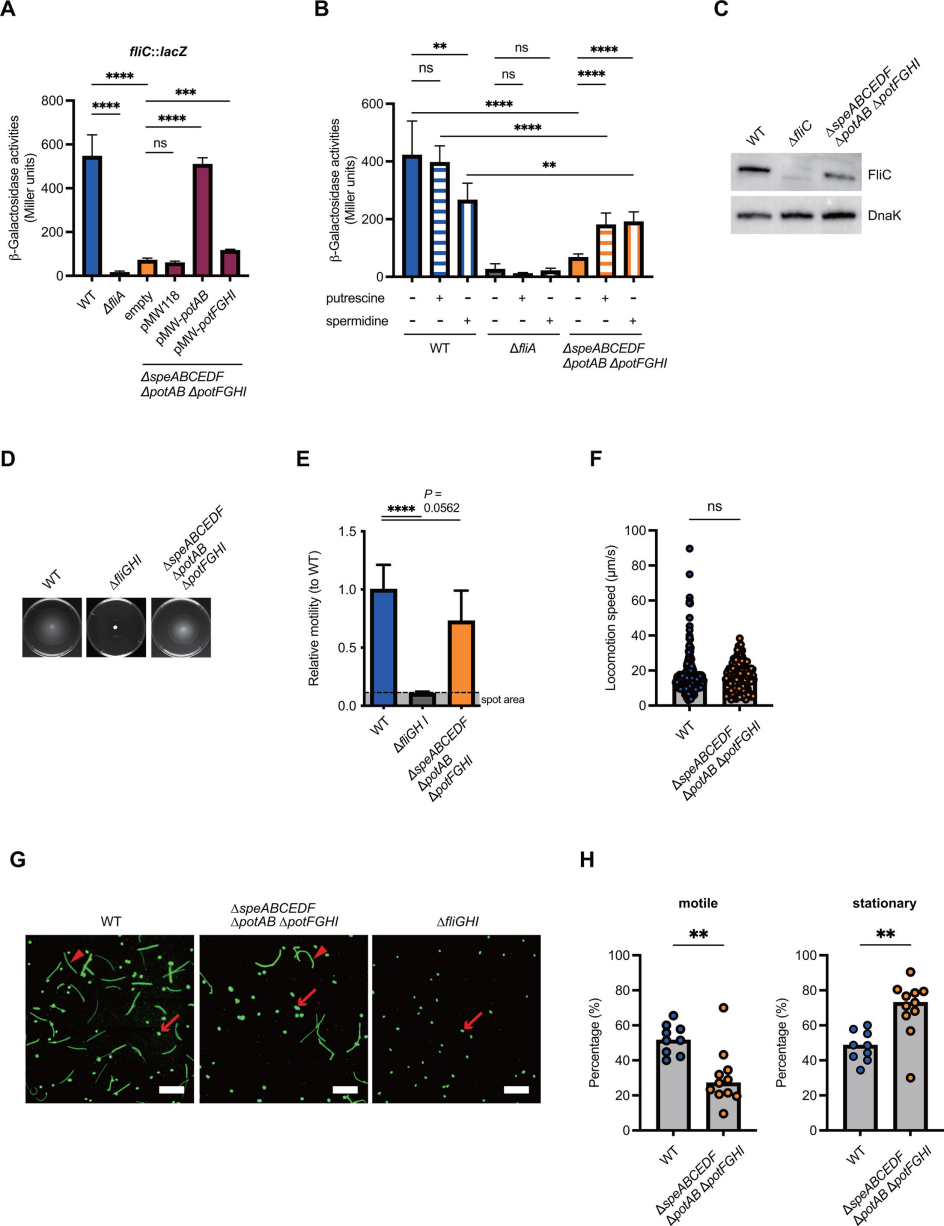
Uptake of polyamines impacts *S*Tm flagellum-driven motility. (**A and B**) Transcription levels of the *fliC* gene of *S*Tm grown in LB medium are presented as units of β-galactosidase activity of the indicated *S*Tm strains. If needed, 3 mM of PUT or SPD was added to the LB medium. Bars represent mean ± SD from at least three independent experiments. A one-way ANOVA followed by Dunnett’s multiple comparisons test. *P* > 0.05 not significant (ns), *P* < 0.05 (*), *P* < 0.01 (**), *P* < 0.001 (***), *P* < 0.0001 (****). (**C**) FliC flagellin expression of *S*Tm WT, ∆*fliC,* and ∆*speABCEDF* ∆*potAB* ∆*potFGHI*. Samples of lysate were subjected to SDS-PAGE and analyzed by western blotting with anti-FliC and anti-DnaK antibodies. DnaK is an internal control. (**D**) Motility on soft-agar plate on *S*Tm WT, ∆*fliGHI,* and ∆*speABCEDF* ∆*potAB* ∆*potFGHI*. (**E**) The relative motility of the experiment in panel D was measured. Data are shown as the means ± standard deviations of the results from three independent experiments. A one-way ANOVA followed by Dunnett’s multiple comparisons test. *P* > 0.05 not significant (ns), *P* < 0.05 (*), *P* < 0.01 (**), *P* < 0.001 (***), *P* < 0.0001 (****). (**F**) The swimming speed of single cells of *S*Tm moving. *n* = 323 [WT], and 162 [∆*speABCEDF* ∆*potAB* ∆*potFGHI*]. Bars, median. Two-tailed Mann‒Whitney U tests were used to compare two groups in each panel. *P* > 0.05 not significant (ns), *P* < 0.05 (*), *P* < 0.01 (**), *P* < 0.001 (***), *P* < 0.0001 (****). (**G**) Representative microscopy images of individual cells expressing green fluorescent protein (GFP) in WT, the ∆*fliGHI,* and the ∆*speABCEDF* ∆*potAB* ∆*potFGHI*. Arrowheads show the motile, and arrows indicate stationary cells. Scale bar, 50 µm. (**H**) Microscopy quantification of *S*Tm motile or stationary cells. Individual data points represent the averages of the single-cell movement behavior (motile or stationary) per high-power fields from independent experiments. Bars, median. Two-tailed Mann‒Whitney U tests were used to compare two groups in each panel. *P* > 0.05 not significant (ns), *P* < 0.05 (*), *P* < 0.01 (**), *P* < 0.001 (***), *P* < 0.0001 (****).

According to our RNA-seq analysis, many genes involved in flagellar motility were downregulated in the ∆*speABCEDF* ∆*potAB* ∆*potFGHI* mutant (Fig. S4). Thus, we investigated whether polyamines affect flagellar gene expression by using *fliC::lacZ* reporter strains. The *fliC* expression levels in the ∆*speABCEDF* ∆*potAB* ∆*potFGHI* mutant were reduced compared with those in the WT, as well as in the ∆*fliA* mutant, which is incapable of transcriptional activation for class 3 flagellar genes such as *fliC* (Fig. 5A). Transformation of plasmids encoding *potABCD* or *potFGHI* restored the expression of *fliC*, indicating that polyamine uptake is involved in flagellar gene expression. The addition of PUT to the WT culture medium had no effect on *fliC* expression, whereas SPD supplementation significantly reduced expression (Fig. 5B). Neither PUT nor SPD affected *fliC* expression levels in the ∆*fliA* mutant. In contrast, the expression levels of *fliC* in the ∆*speABCEDF* ∆*potAB* ∆*potFGHI* mutant increased with supplementation of either PUT or SPD (Fig. 5B). It is notable that *fliC* expression levels in SPD-supplemented WT and the ∆*speABCEDF* ∆*potAB* ∆*potFGHI* mutant were similar, but significantly different. Furthermore, the reduced expression of FliC protein in the ∆*speABCEDF* ∆*potAB* ∆*potFGHI* mutant was confirmed by western blot analysis using anti-FliC antibodies (Fig. 5C). These results indicate that polyamines are involved in flagellar biogenesis by activating the expression of flagellar genes.

Next, we examined whether the reduced expression of flagellar genes affects flagellum-based motility in the ∆*speABCEDF* ∆*potAB* ∆*potFGHI* mutant. *S*Tm WT cells spread concentrically on semi-agar (0.3% agar) LB medium, whereas the ∆*fliGHI* mutant, which lacks flagella, was non-motile (Fig. 5D and E). In contrast, the ∆*speABCEDF* ∆*potAB* ∆*potFGHI* strain showed a slight reduction in flagellum-driven motility compared with the WT. Moreover, the swimming speed of the motile cells in the ∆*speABCEDF* ∆*potAB* ∆*potFGHI* strain was equivalent to that of the WT, as evidenced by the statistical analysis (*P* = 0.53; median WT: 15.7 µm/s, ∆*speABCEDF* ∆*potAB* ∆*potFGHI*: 16.6 µm/s) (Fig. 5F). However, the WT had a higher proportion of motile cells than the ∆*speABCEDF* ∆*potAB* ∆*potFGHI* strain, whereas the proportion of stationary cells was higher in the ∆*speABCEDF* ∆*potAB* ∆*potFGHI* compared with the WT (Fig. 5G and H). All control cells from the ∆*fliGHI* mutant were stationary (Fig. 5G). Our findings highlight that although impaired polyamine homeostasis does not significantly impact the motor activity driven by flagella, it does influence the population balance between motile and nonmotile cells in *S*Tm. In summary, our data underscore the importance of uptake-dependent polyamine homeostasis for flagellum-based movement in individual *S*Tm cells, as it supports the expression of flagellar genes.

### Polyamine homeostasis‒dependent nitrate respiration and flagellar motility are involved in *S*Tm gut colonization

**Fig 6:**
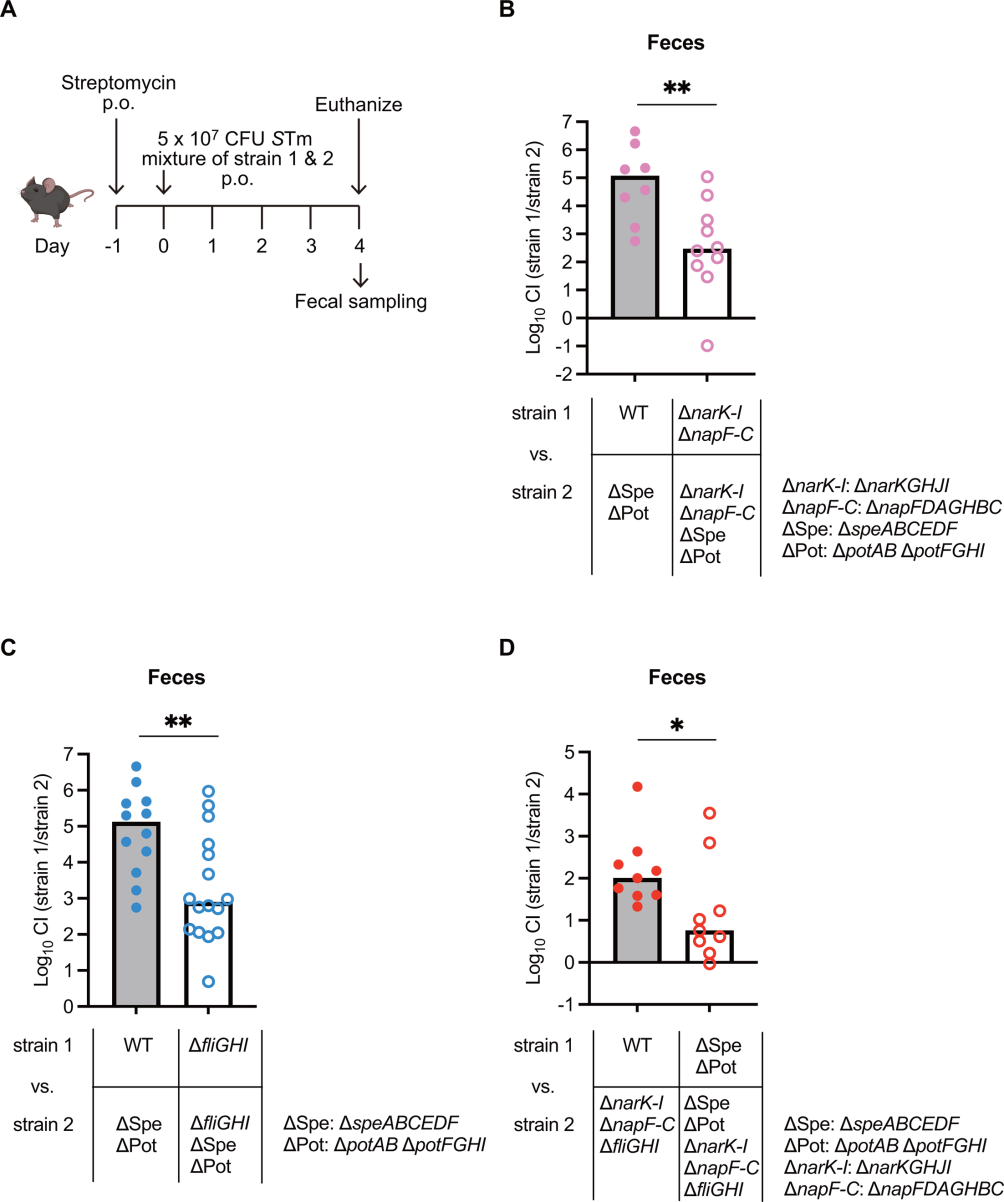
Nitrate respiration and flagellar motility are involved in polyamine homeostasis‒dependent gut colonization. (**A**) Experimental scheme of *S*Tm oral infection in the streptomycin-treated mouse model. C57BL/6 SPF mice were pre-treated with 25 mg of streptomycin by oral gavage 24 h before oral infection with *S*Tm (1:1 mixture of strain 1 and strain 2). Mice were euthanized on day 4 post-infection (4 dpi), and feces were collected. (**B through D**) The CI of *S*Tm loads recovered from the feces was determined by selective plating. Bars, median. Two-tailed Mann‒Whitney U tests were used to compare two groups in each panel. *P* > 0.05 not significant (ns), *P* < 0.05 (*), *P* < 0.01 (**), *P* < 0.001 (***), *P* < 0.0001 (****).

The ∆*speABCEDF* ∆*potAB* ∆*potFGHI* strain demonstrated reduced gut colonization compared with the WT (44). Moreover, the involvement of nitrate respiration in *S*Tm expansion within the gut lumen is known (8, 48, 49). To investigate the potential involvement of nitrate respiration in polyamine homeostasis‒dependent gut colonization, we employed the ∆*narKGHJI* ∆*napFDAGHBC* genetic background strains that cannot colonize in the nitrate respiration-dependent fashion. First, we examined the competitive colonization levels of the ∆*narKGHJI* ∆*napFDAGHBC* strain, which is incapable of nitrate respiration (Fig. 6A). Consistent with previous research (48, 49), the ∆*narKGHJI* ∆*napFDAGHBC* strain lacking nitrate respiration activity was impaired in gut colonization in a gastrointestinal infection mouse model (Fig. S5).

We next performed mouse infection experiments using the ∆*narKGHJI* ∆*napFDAGHBC* ∆*speABCEDF* ∆*potAB* ∆*potFGHI* strains. In feces, the CI values of the ∆*narKGHJI* ∆*napFDAGHBC* strain compared with those of the ∆*narKGHJI* ∆*napFDAGHBC* ∆*speABCEDF* ∆*potAB* ∆*potFGHI* strain were significantly reduced compared with the WT versus ∆*speABCEDF* ∆*potAB* ∆*potFGHI* (Fig. 6B), indicating that the reduced expression of the *narKGHJI* and *napFDAGHBC* operons was at least partly responsible for the attenuated gut colonization of the ∆*speABCEDF* ∆*potAB* ∆*potFGHI* strain. These results suggest that nitrate respiration mediated by the NarGHI and NapABC protein complexes is involved in the polyamine-dependent gut colonization of *S*Tm.

Next, we investigated whether the reduced flagellum-driven motility caused by impaired polyamine homeostasis influences *S*Tm virulence. We employed a mouse model for gastrointestinal infection by *S*Tm and performed CI experiments using the non-motile ∆*fliGHI* background strains. The CI values in feces for the ∆*fliGHI* strain compared with the ∆*fliGHI* ∆*speABCEDF* ∆*potAB* ∆*potFGH* strain were lower than those for the WT compared with the ∆*speABCEDF* ∆*potAB* ∆*potFGHI* strain (Fig. 6C). These results indicate that the reduced flagellar motility of the ∆*speABCEDF* ∆*potAB* ∆*potFGHI* strain is partly responsible for the impaired luminal growth of *S*Tm.

Next, to better define the causal link between polyamine homeostasis‒dependent gut colonization and flagellum-driven motility/nitrate respiration, we compared the CI values between the WT and the ∆*narKGHJI* ∆*napFDAGHBC* ∆*fliGHI* mutant and between the ∆*speABCEDF* ∆*potAB* ∆*potFGHI* mutant and the ∆*speABCEDF* ∆*potAB* ∆*potFGHI* ∆*narKGHJI* ∆*napFDAGHBC* ∆*fliGHI* mutant. CI values in the feces for the WT versus ∆*narKGHJI* ∆*napFDAGHBC* ∆*fliGHI* mutant comparison were further decreased upon introduction of the ∆*speABCEDF* ∆*potAB* ∆*potFGHI* genetic background (Fig. 6D). These results suggest that polyamine homeostasis acts as a colonization factor, similar to flagellar motility and nitrate respiration.

**Fig 7:**
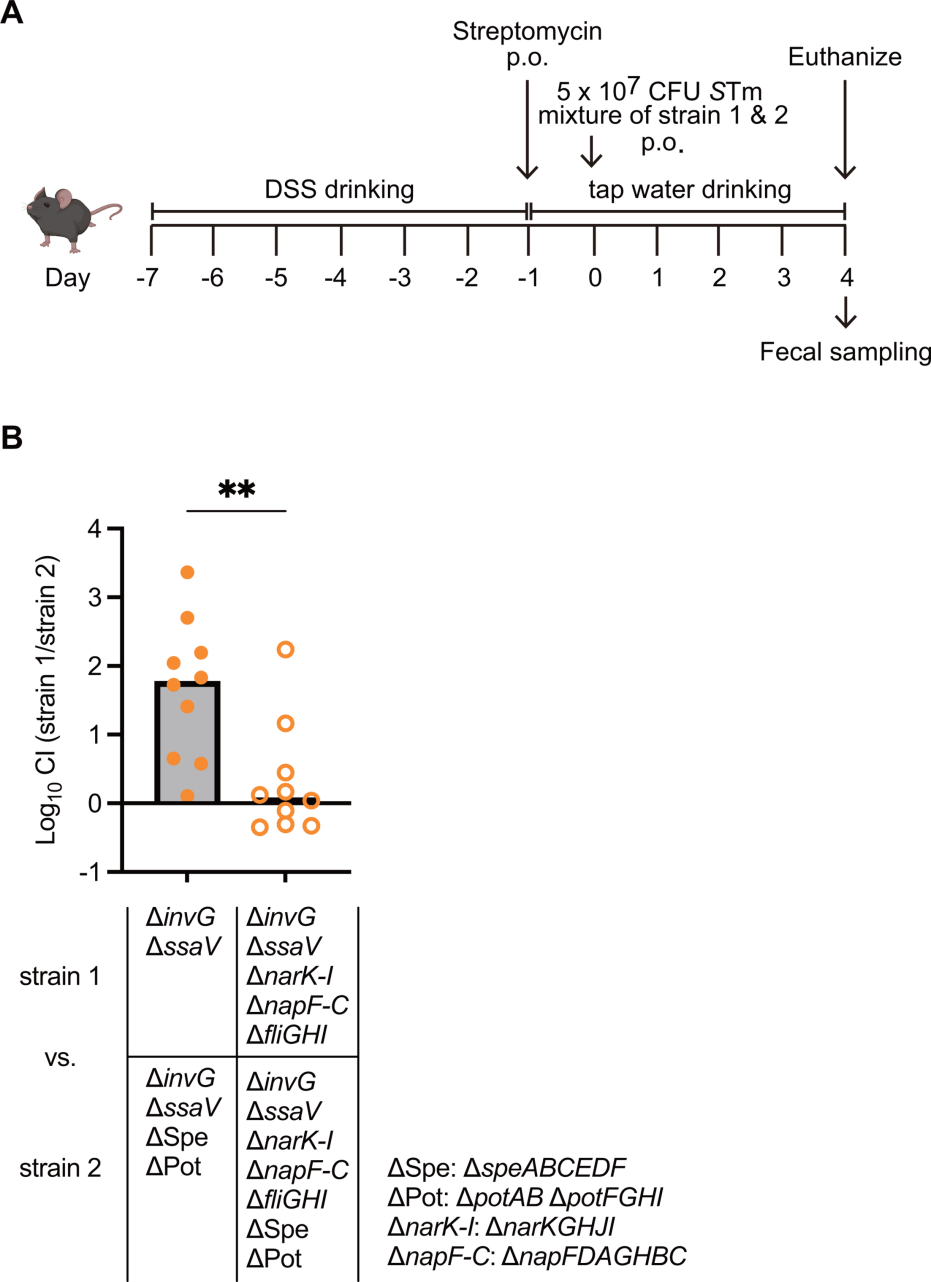
Nitrate respiration and flagellar motility are required for polyamine homeostasis‒dependent gut colonization, independently of T3SS. (**A**) Experimental scheme of *S*Tm oral infection in the streptomycin-treated dysbiotic mouse model. Two percent of DSS-drinking C57BL/6 SPF mice were pre-treated with 25 mg of streptomycin by oral gavage 24 h before oral infection with *S*Tm (1:1 mixture of strain 1 and strain 2). Mice were euthanized on day 4 post-infection, and the feces were collected. (**B**) The CI of *S*Tm loads recovered from the feces was determined by selective plating. Bars, median. Two-tailed Mann‒Whitney U tests were used to compare two groups in each panel. *P* > 0.05 not significant (ns), *P* < 0.05 (*), *P* < 0.01 (**), *P* < 0.001 (***), *P* < 0.0001 (****).

Recent reports indicate that host polyamine uptake‒dependent homeostasis is crucial to the two distinct T3SS‒dependent virulence mechanisms in *S*Tm (44). Both T3SSs are necessary for sustained colonization in the gut, primarily by inducing gut inflammation, as evidenced by the failure of the T3SS-deficient mutant (∆*invG* ∆*ssaV*) to colonize at higher levels on day 4 post-infection compared with the WT (Fig. S6A and B). Furthermore, our previous work demonstrated that the failure to induce gut inflammation by infection with this mutant strain suggests that sustained gut colonization by the ∆*invG* ∆*ssaV* mutant requires inflammatory responses (50). To clarify whether the polyamine homeostasis contributes to gut colonization of *S*Tm independently of the T3SSs, we investigated the gut colonization levels of the ∆*invG* ∆*ssaV* genetic background strains in a dextran sulfate sodium (DSS) mouse model, which we employed to recapitulate gut inflammation (Fig. 7A). CI values of the *∆invG ∆ssaV ∆narKGHJI napFDAGHBC ∆fliGHI* versus the *∆invG ∆ssaV ∆narKGHJI napFDAGHBC ∆fliGHI ∆speABCEDF ∆potAB ∆potFGHI* were significantly reduced compared with those of the *∆invG ∆ssaV* versus the ∆*invG* ∆*ssaV ∆speABCEDF ∆potAB ∆potFGHI* (Fig. 7B). These results show that the impaired colonization of the *∆speABCEDF ∆potAB ∆potFGHI* is due to the activities of nitrate respiration and flagellar motility, independently of T3SS. Collectively, our data demonstrate that the polyamine homeostasis‒dependent activities of nitrate respiration and flagellar motility contribute to the pathogenesis of gut infection by *S*Tm.

### Intestinal luminal polyamines enhance the gut colonization by *S*Tm and a pathobiont *E*. *coli*

**Fig 8:**
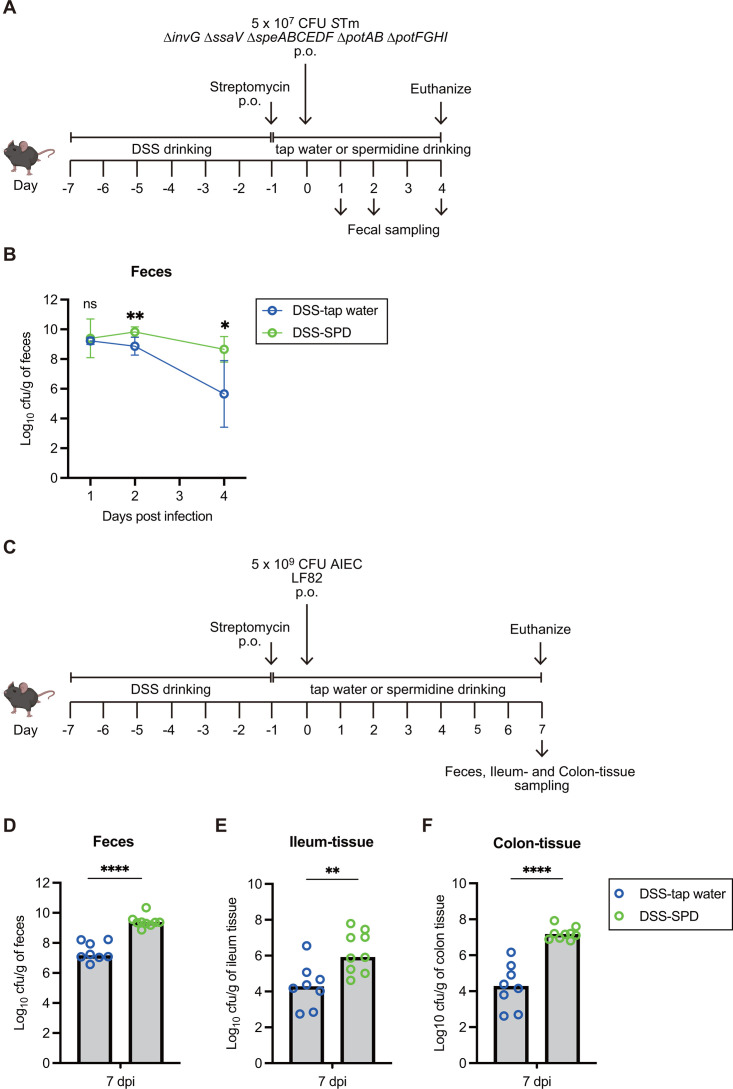
Luminal spermidine improves *S*Tm lacking the T3SSs and *E. coli* fitness in the intestinal tract. (**A**) Experimental scheme of *S*Tm oral infection in the streptomycin-treated dysbiotic mouse model. Two percent of DSS-drinking C57BL/6 SPF mice were pre-treated with 25 mg of streptomycin by oral gavage 24 h before oral infection with *S*Tm ∆*invG* ∆*ssaV* ∆*speABCEDF* ∆*potAB* ∆*potFGHI* mutant (an infectious dose of 5 × 10^7^). Mice were treated with 1% spermidine (SPD) in the drinking water and euthanized on day 4 post-infection (4 dpi). On days 1, 2, and 4 post-infection, the feces were collected, and the bacterial loads recovered from the feces were determined by selective plating. (**B**) The fecal burden of *S*Tm was monitored for 4 days post-infection. Data points represent means ± SD. Two-tailed Mann‒Whitney U tests to compare two groups in each panel. *P* > 0.05 not significant (ns), *P* < 0.05 (*), *P* < 0.01 (**), *P* < 0.001 (***), *P* < 0.0001 (****). (**C**) Experimental scheme of AIEC oral infection in the streptomycin-treated DSS mouse model. Two percent of DSS-drinking C57BL/6 SPF mice were pre-treated with 25 mg of streptomycin by oral gavage 24 h before oral infection with AIEC strain LF82 (an infectious dose of 5 × 10^9^). Mice were euthanized on day 7 post-infection (7 dpi), and the feces, ileum tissues, and colon tissues were collected. (**D through F**) Bacterial loads recovered from feces (**D**), ileum tissues (**E**), and colon tissues (**F**) were determined by selective plating. Bars, median. Two-tailed Mann‒Whitney U tests to compare two groups in each panel. *P* > 0.05 not significant (ns), *P* < 0.05 (*), *P* < 0.01 (**), *P* < 0.001 (***), *P* < 0.0001 (****).

Finally, to demonstrate the link between luminal polyamine contents and the gut colonization of *S*Tm, we explored whether SPD supplementation to DSS model mice affects the colonization levels of *S*Tm (Fig. 8A). In feces, the colonization levels of the ∆*invG* ∆*ssaV ∆speABCEDF ∆potAB ∆potFGHI* mutant in tap water‒drinking mice gradually decreased over the course of the infection (Fig. 8B). In contrast, the ∆*invG* ∆*ssaV ∆speABCEDF ∆potAB ∆potFGHI* mutant persistently colonized the gut lumen of SPD‒drinking mice. These results suggest that intestinal luminal contents of polyamines correlate with levels of *S*Tm gut colonization by maintaining polyamine homeostasis. Furthermore, this is independent of T3SS activity, as seen in the ∆*invG* ∆*ssaV* genetic background. Based on previous results in this study, we predict that luminal SPD involves the activities of nitrate respiration and flagellar motility, at least in part by maintaining polyamine homeostasis, leading to improved fitness of *S*Tm in the gut lumen.

Moreover, we investigated whether increased luminal SPD could impact the gut colonization of the *S*Tm strain while maintaining polyamine homeostasis. In the feces from DSS model mice, the colonization levels of the ∆*invG* ∆*ssaV* mutant in SPD‒drinking mice were higher than those in tap water‒drinking mice (Fig. S7A and B). The results suggest that luminal polyamines enhance the gut colonization of *S*Tm while still maintaining polyamine homeostasis, probably due to enhanced gene expressions of flagellar motility and nitrate respiration at least in part.

We next considered whether intestinal luminal polyamines promote the growth of other enteric bacterial pathogens besides *S*Tm. We employed a pathobiont *Escherichia coli* (an adherent-invasive *E. coli*, AIEC) that lacks T3SS but can colonize the gut lumen and invade intestinal tissues in a DSS mouse model (Fig. 8C). Similar to the case with *S*Tm, the fecal colonization levels of AIEC in SPD-drinking mice were higher than those in tap water‒drinking mice (Fig. 8D). Similar results were observed in the ileum and colon tissue (Fig. 8E and F). These findings indicate that luminal polyamines not only support *S*Tm growth but also that increased luminal SPD can enhance the expansion of another enteric pathogen, AIEC. Although future studies are needed to decipher the molecular mechanism underlying the enhanced colonization of AIEC, luminal polyamine‒dependent expansion may be generalized to enteric bacterial pathogens.

## DISCUSSION

Gut microbiota-derived metabolites influence the outcomes of gastrointestinal infections. Our findings raise the possibility that colonic luminal SPD drives gut colonization by enteric bacterial pathogens. An *S*Tm strain lacking genes associated with polyamine synthesis and uptake exhibited impaired ability to colonize the intestinal tract, attributed to reduced expression of genes involved in flagellar motility and nitrate respiration. Furthermore, SPD-enhanced expansion in the luminal gut was observed in both *S*Tm and a pathobiont *E. coli*. Therefore, we propose that a scenario in which specific members of the microbiota produce higher luminal levels of SPD may be a risk factor for infections with enteric bacterial pathogens (Fig. S8).

Polyamines participate in the transcriptional regulation of genes at the post-transcriptional level by forming a polyamine–RNA complex (51). Our transcriptome analysis revealed previously unidentified polyamine-dependent genes that influence the gut colonization of *S*Tm, including those associated with flagellar motility and nitrate respiration. Flagellar motility, encompassing bacterial locomotion and chemotaxis, is crucial for *Salmonella* gut infection, as it propels the pathogen toward the intestinal epithelium, thereby accelerating disease progression (3, 11). Our data indicate that uptake-dependent polyamine homeostasis affects the expression of many genes involved in flagellar motility and chemotaxis (52). We speculate that the decline in flagellar gene expression is due to lower transcript levels of *flhDC*, which encodes master regulator proteins, and of *fliA*, which is responsible for an RNA polymerase sigma factor that regulates class 3 flagellar genes, as evidenced by our RNA-seq and RT-qPCR findings. Notably, a recent study showed that SPD is involved in FliA expression (42). Unexpectedly, however, we found that the swimming motility of the ∆*speABCEDF* ∆*potAB* ∆*potFGHI* strain in soft agar was reduced very slightly compared with that of the WT. Furthermore, the swimming velocities of motile cells were similar between the WT and the ∆*speABCEDF* ∆*potAB* ∆*potFGHI* strains. In contrast, the proportion of stationary cells in the ∆*speABCEDF* ∆*potAB* ∆*potFGHI* strain was increased compared with the WT. It is thus tempting to speculate that impaired polyamine homeostasis and the resultant reduced expression levels of flagellar genes may contribute to an increased population of *S*Tm cells incapable of flagellum-driven movement, likely due to insufficient flagellum construction. Finally, using an *fliGHI*-deficient genetic background, our data revealed that the altered population balance of motile and nonmotile *S*Tm cells influences polyamine-dependent gut colonization of *S*Tm. These findings provide new insights into flagellum-mediated gut infection by *S*Tm. We propose that luminal levels of polyamines may influence infection efficiency by contributing to cooperative flagellum-driven motility.

Our data indicate that the functioning of operon genes, such as *narGHI* and *napABC*, encoding nitrate reductase complexes, relies on polyamines. The ∆*speABCEDF* ∆*potAB* ∆*potFGHI* strain displayed impaired nitrate respiration activities, similar to the mutants lacking *narGHI* and *napABC*. Consistent with earlier studies showing that nitrate respiration enhances *S*Tm gut colonization (48, 49, 53), we show that the reduced colonization of the ∆*speABCEDF* ∆*potAB* ∆*potFGHI* strain is at least partly attributable to reduced expression of nitrate reductase. The state of intestinal inflammation during pathogen infection, antibiotic treatment, or inflammatory bowel disease (IBD) leads to increased nitrate production (54–57). Notably, the expansion of Enterobacteriaceae correlates with increased nitrate levels (58, 59). Indeed, gut inflammation raises the availability of nitrate, providing a nutrient niche for *S*Tm or *E. coli*, whose luminal growth is supported by nitrate, acting as an electron acceptor (57, 58). Therefore, we propose a novel axis—polyamine homeostasis and nitrate respiration—that allows enteric pathogens to exploit increased luminal nitrate for expansion. Targeting polyamine homeostasis in enteric pathogens could inhibit their expansion in the inflamed gut. Furthermore, reducing luminal polyamine levels may offer a new therapeutic approach for IBD.

Here, we demonstrate that SPD is necessary for maintaining *napFDAGHBC* operon expression levels. The results from the RNA-seq, β-galactosidase assays, and RT-qPCR suggest that SPD stimulates *napF* promoter activity in *S*Tm. The expression of the *napFDAGHBC* operon is known to be regulated by two two-component systems: NarXL and NarPQ (60). NarP upregulates the *napFDAGHBC* operon, whereas NarL inhibits transcription activity under high nitrate concentrations. Thus, we tried to clarify whether NarP is involved in the polyamine-dependent expression of the *napFDAGHBC* operon. Adding SPD to the culture medium did not increase the expression of *napF* in the ∆*narP* mutant. Moreover, overexpression of NarP did not restore *napF* expression in the ∆*speABCEDF* ∆*potAB* ∆*potFGHI* strain. Finally, we found that the activities of *narP* expression and the binding of NarP to the *napF* promoter were similar between the *S*Tm WT and the ∆*speABCEDF* ∆*potAB* ∆*potFGHI* strains. In line with previously reported examples of polyamine-dependent gene expression (51, 61), in which SPD stimulates the synthesis of specific proteins at the levels of translation by binding transcribed mRNA, our present findings indicate that SPD activates the expression of the *napFDAGHBC* operon post-transcriptionally in *S*Tm, where SPD probably influences translation efficiency by binding the *napF* mRNA.

Previously, we showed that *S*Tm infection leads to increased levels of host polyamines in the intestinal tract and spleen (44). This is part of the host inflammatory response, as evidenced by the elevated polyamine levels found in *S*Tm-infected mice and a DSS-treated dysbiotic mouse model. That study also revealed that elevated levels of intracellular polyamines result from heightened arginase activity (44). However, the source of luminal polyamines in response to *S*Tm infection remains unclear. As previously mentioned, this is involved in the host’s inflammatory response, but we do not yet know the source. Based on our results that inflammation-induced polyamines fuel the luminal growth of enteric bacterial pathogens, including *S*Tm and AIEC, inhibiting inflammation-associated polyamine production may lead to new therapeutic interventions for infections caused by enteric bacterial pathogens.

## MATERIALS AND METHODS

### Bacterial strains and culture conditions

The WT *S*Tm strain SL1344 (62) and its derivative strains were used in this study (Table S3). Chromosomal in-frame deletion mutants of SL1344 were generated using the Lambda Red recombination system (63) or through P22 phage-mediated transduction as described below. All bacterial strains were routinely cultured at 37°C overnight in Luria‒Bertani (LB) broth with agitation or on LB agar supplemented with antibiotics as needed, including streptomycin (50 µg/mL), chloramphenicol (10 µg/mL), kanamycin (50 µg/mL), or ampicillin (100 µg/mL). When necessary, bacteria were cultivated in M9 minimal medium (47.7 mM Na_2_HPO_4_, 22 mM KH_2_PO_4_, 8.5 mM NaCl, 18.7 mM NH_4_Cl, 1 mM MgSO_4_, 0.1 mM CaCl_2_, 0.1% glucose, and 0.01% histidine) with appropriate antibiotic supplementation.

Additionally, *E. coli* strain LF82 (64), used in this study, was cultured under the same conditions on agar plates and in broth, similar to the *S*Tm strains.

### Mice

C57BL/6 mice were housed under specific pathogen-free (SPF) conditions at the animal research facilities of the School of Pharmacy, Kitasato University. When necessary, additional SPF C57BL/6 mice were acquired from Japan SLC. All animals were maintained on CE-2 standard rodent chow (CLEA Japan) and allowed to acclimate to their housing environment to ensure uniform microbiota. All animal procedures were conducted in compliance with ethical regulations and were approved by the Kitasato University Institutional Animal Care and Use Committee (Permit Number: 24-8). Male and female mice, aged 6–12 weeks, were included in the experiments to mitigate potential sex-based variability. Mice were randomly allocated to experimental groups, and no significant sex-associated phenotypic differences were observed across repeated trials.

### Generation of *S*Tm mutant strains

Isogenic deletion mutants of SL1344 were generated using the Lambda Red recombinase system following established protocols (63). Primers were designed with 40 flanking base pairs of the gene of interest and 20 base pairs of the chloramphenicol or kanamycin resistance cassette from pKD3 or pKD4. To amplify the DNA fragment, including the antibiotic resistance cassette flanked by the regions of the gene of interest, PCR was then performed using pKD3 or pKD4 as a DNA template. The Monarch PCR & DNA cleanup kit (New England Biolabs) or the FastGene gel/PCR extraction kit (NIPPON Genetics) was used to purify the PCR products. *S*Tm carrying pKD46 was cultured in 50 mL of LB medium supplemented with ampicillin and 10 mM L-arabinose at 30°C for 3 h. After washing with ice-cold dH_2_O, the bacterial cells were concentrated by resuspension in 10% glycerol. Following electroporation at 1.8 kV for 5 ms, 5 µL of the purified PCR products was introduced into the cells, which were then incubated in warm LB at 37°C with shaking for 1.5 h before being plated on LB agar containing 10 µg/mL chloramphenicol or 50 µg/mL kanamycin. The colonies were isolated on LB plates without antibiotics and incubated at 42°C to eliminate pKD46. PCR screening verified the desired mutations, followed by P22 phage-mediated transduction into SL1344. Additional rounds of P22 transduction were conducted to generate double or multiple gene-disrupted mutants.

### Mouse infections

The streptomycin mouse model was used as previously described (65–67). Mice received an oral dose of 25 mg streptomycin 24 h before infection. For infection, 5 × 10^7^ CFU of *S*Tm or an equal mixture of bacterial cultures in co-infection experiments (totaling 1 × 10^8^ CFU) was administered via oral gavage. Alternatively, 5 × 10^7^ CFU of *S*Tm or 5 × 10^9^ CFU of AIEC of bacterial cultures was infected by oral gavage. To recapitulate the gut inflammation, mice were administwith a dose of 2% (wt/vol) DSS (MW, 5,000 Da; FUJIFILM Wako Pure Chemical Corporation) in drinking water. To prevent cross-contamination, the mice were housed in cages with mesh flooring. To evaluate *S*Tm colonization, feces were collected and homogenized in sterile phosphate-buffered saline (PBS). Homogenization was carried out using a tissue lyser device (Qiagen) set at 25 Hz for 2 min. The resulting homogenates were serially diluted in PBS and then plated on LB agar containing the appropriate antibiotic(s) for selective growth. Colonies were enumerated following overnight incubation at 37°C. The population sizes of *S*Tm or AIEC were recorded as CFU per gram of feces or as CFU per organ (ileum and colon). The competitive index (CI) was determined by calculating the ratio of *S*Tm parent strain populations to their corresponding mutant derivatives and normalizing the ratio to the initial inoculum.

### Determination of intracellular contents of polyamines in bacteria

Bacteria grown up to the middle-logarithmic growth phase in LB were centrifuged at 16,000 × *g* for 5 min at 4°C, and the pellet was washed with PBS. The pellet was resuspended in 300 µL of 5% (vol/vol) trichloroacetic acid (FUJIFILM Wako Pure Chemical Corporation) and boiled for 15 min, followed by standing on ice for 15 min. After centrifugation at 16,000 × *g* for 5 min at 4°C, the supernatant was filtered with a Cosmonice Filter W (Nacalai Tesque) and subjected to an HPLC system (Chromaster, Hitachi) equipped with a cation exchange column (#2619 PH, 4.6 × 50 mm, Hitachi) for the analysis of polyamine concentration, as described previously (45).

### RNA isolation from bacteria and reverse-transcription quantitative real-time PCR

Bacterial cultures grown in LB or M9 media were separated from the medium via centrifugation, and total RNA was extracted using the Direct-zol RNA MiniPrep kit (Zymo Research) following the manufacturer’s protocol. RNA samples were stored at 80°C, and their concentrations and purity levels were assessed using a NanoDrop 1000 spectrophotometer (Thermo Fisher Scientific). Reverse transcription was conducted using TaqMan Reverse Transcription reagents (Invitrogen). Quantitative real-time PCR (qPCR) using SYBR Fast qPCR master mix (Kapa Biosystems) was conducted on a CFX96 real-time PCR detection system (Bio-Rad) to amplify the target genes. Table S4 lists the primer sequences used for amplification. Relative transcript levels were normalized to the *rpoD* gene and calculated using the 2^-∆^*^CT^* method (68).

### Generation of RNA sequencing data

Independent triplicate cultures of *S*Tm ∆*speABCEDF* and ∆*speABCEDF* ∆*potAB* ∆*potFGHI* were cultivated in LB medium at 37°C until reaching mid-log phase. Total RNA was extracted from the collected bacterial cells using the Direct-zol RNA MiniPrep kit (Zymo Research) in accordance with the manufacturer’s instructions. Strand-specific RNA libraries were then constructed using the NEBNext Ultra Directional RNA Library Prep kit for Illumina (New England BioLabs). Sequencing was performed on the Illumina Novaseq 6000 platform to generate high-throughput transcriptomic data.

### RNA-seq analysis

Quality sequencing reads were aligned to the *S*Tm strain SL1344 genome (NCBI reference sequence with GenBank accession number NC_016810.1) using HISAT2 v2.1.0 (http://daehwankimlab.github.io/hisat2/) (69). Differential expression genes (DEGs) analysis was conducted with the DESeq2 package v1.24.0 (https://bioconductor.org/packages/release/bioc/html/DESeq2.html) (70). Genes with a *P* value of < 0.05, an adjusted *P* value of < 0.01, and a fold change using a default cutoff greater than or equal to ±2.0 were considered significantly differentially expressed. PCA was generated using the ggplot2 v3.0.1.1 (https://cran.r-project.org/web/packages/ggplot2/index.html) (71). Heat maps were plotted using Heatmapper (http://www.heatmapper.ca) (72).

### Construction of chromosomal transcriptional *lacZ* reporter strains

*S*Tm *napF*, *narP*, or *fliC* promoter regions were amplified by PCR using the primer sets SL Pro-napF-SalI and SL Rev-napF-BamHI, SL Pro-narP-SalI and SL Rev-narP-BamHI, or fliC-Pro-SalI and fliC-Rev-BamHI, respectively (Table S4). The PCR products were digested with SalI and BamHI, then ligated into the same sites of pLD-*lacZ*Ω containing a promoterless *lacZ* gene (73), yielding pLD-*napFZ* or pLD-*narPZ* or pLD-*fliCZ. E. coli* SM10λ*pir* was transformed by the resulting integration plasmid and transferred to the *S*Tm strain SH100 (73) by conjugation. The *napF::lacZ*, *narP::lacZ*, or *fliC::lacZ* allele was transduced into SL1344 through the P22 phage.

### Construction of a plasmid expressing NarP and 6xHis-tagged NarP protein

To generate plasmids for the expression of NarP with a 6× His tag, the narP gene from *S*Tm was amplified via PCR. Specific primer pairs—SL narP-KpnI-FW and SL narP-SalI-RV or SL narP-XhoI-FW and SL narP-EcoRI-RV (Table S4)—were used for amplification. The resulting PCR products were digested with either KpnI and SalI or XhoI and EcoRI restriction enzymes. These digested DNA fragments were subsequently ligated into the corresponding sites of the expression vectors pMW118 or pBAD/His A, yielding the recombinant plasmids pMW-*narP* or pBAD-*narP*.

### β-Galactosidase assays

β-Galactosidase activity was assayed following established protocols (50). Briefly, bacterial cultures were incubated overnight in LB medium, diluted 1:100 in 5 mL of fresh LB, and further cultured at 37°C for 3 h. A 100 µL aliquot of the bacterial suspension was combined with 900 µL of Z buffer (60 mM Na_2_HPO_4_·7H_2_O, 40 mM NaH_2_PO_4_·H_2_O, 10 mM KCl, 1 mM MgSO_4_·7H_2_O, 50 mM β-mercaptoethanol). Subsequently, 20 µL of 0.1% (wt/vol) SDS and 40 µL of chloroform were added, and the mixture was thoroughly vortexed. After incubation at 28°C for 5 min, the reaction was initiated by adding 200 µL of *o*-nitrophenyl-β-D-galactopyranoside (ONPG) (4 mg/mL) in 0.1 M potassium phosphate buffer (pH 7). The reaction proceeded at 28°C until the color changed. To terminate the reaction, 500 µL of 1 M Na_2_CO_3_ was introduced, and absorbance at 420 nm (OD_420_) was recorded. β-Galactosidase activity was expressed in Miller units, calculated as described previously (50).

### Chromatin immunoprecipitation (ChIP) analysis

*S*Tm WT and ∆*speABCEDF* ∆*potAB* ∆*potFGHI* expressing His-tagged NarP were cultured in LB to an OD_600_ of 1.0 and then incubated with 1% formaldehyde at room temperature for 15 min to cross-link DNA with protein. After the incubation, glycine was added to reach a final concentration of 125 mM to quench the reaction. Bacteria were harvested, washed twice in cold PBS, and osmotically lysed. Genomic DNA was then sheared by sonication to an average size of 300 bp and incubated with ChIP-grade protein G magnetic beads (Cell Signaling Technology) to pre-clean the chromatin. The resulting lysates were incubated with anti-His mAB (clone: OGHis) (MBL) at 4°C for 4 h with rotation and immunoprecipitated using ChIP-grade protein G magnetic beads (Cell Signaling Technology). Elutes were incubated with RNase A (10 mg/mL) and proteinase K (20 mg/mL) to reverse the cross-linking. The precipitated genomic DNA fragments from the ChIP samples were purified using a DNA purification kit. For qPCR, ChIP and input samples were analyzed using a CFX96 real-time PCR detection system (Bio-Rad) under standard cycle conditions for SYBR Fast qPCR master mix (Kapa Biosystems). The degree of enrichment for WT was calculated relative to the ∆*speABCEDF* ∆*potAB* ∆*potFGHI* and normalized to the internal control *dnaK*.

### Nitrate reductase assay

*S*Tm was grown overnight in LB, diluted to 1:100, and subcultured for 3 h in LB or LB containing 40 mM nitrate. The supernatant was harvested by centrifugation and subjected to NO_2_/NO_3_ assay kit-C II (Colorimetric) and the Griess reagent kit (DOJINDO) following the manufacturer’s instructions.

### Analysis of flagellin production

The bacterial pellet grown in LB was resolved in SDS-PAGE sample buffer and analyzed by SDS-PAGE and western blotting with anti-*Salmonella* type H-I serum (Denka Seiken Co., Ltd.) or anti-DnaK antibody (Abcam).

### Motility assay with soft agar plate

*S*Tm was grown up to the late logarithmic growth phase in LB broth. A 5 µL aliquot at an OD_600_ of 0.6 was placed on a 0.3% agar LB plate and incubated at 37°C for 8 h.

### Analysis of flagellar motility in liquid culture with microscopy

Analysis for bacterial swimming with microscopy was performed as previously described (74). *S*Tm strains grown in LB broth supplemented with 0.5% glucose were placed on a glass slide and sealed under a glass coverslip. The samples were observed and imaged with an exposure time of 2.6 s with a Zeiss Axio Vert.A1 microscope. Swimming behavior (motile and stationary cells) was evaluated by visual monitoring.

### Statistical analysis

All statistical analyses were conducted using GraphPad Prism 10 for macOS (GraphPad Software). The choice of statistical test was based on the data set characteristics, with one-way ANOVA followed by Dunnett’s multiple comparisons test, the Mann‒Whitney U test, the one-sided Wilcoxon matched-pairs signed rank test, or an unpaired Student’s *t*-test applied as appropriate. Statistical significance was defined as follows: *, *P* < 0.05; **, *P* < 0.01; ***, *P* < 0.001; ****, *P* < 0.0001.

## Data Availability

The raw RNA-seq data discussed in this study have been deposited at the DNA Data Bank of Japan (DDBJ) and are publicly available under accession number PRJDB12861.

## SUPPLEMENTAL MATERIAL

Supplemental material mbio.01786-25-s0001.pdf Supplemental tables and figures.

ASM does not own the copyrights to Supplemental Material that may be linked to, or accessed through, an article. The authors have granted ASM a non-exclusive, world-wide license to publish the Supplemental Material files. Please contact the corresponding author directly for reuse.
