# Supplementary material for: Intestinal luminal polyamines support the gut colonization of enteric bacterial pathogens by modulating flagellar motility and nitrate respiration
Source: mBio. 2025 Aug 11;16(9):e01786-25. doi: 10.1128/mbio.01786-25 (PMC12421874; doi:10.1128/mbio.01786-25)
Supplement: Supplemental material — Supplemental tables and figures. [file mbio.01786-25-s0001.pdf]

SUPPLEMENTAL MATERIAL

Intestinal luminal polyamines support the gut colonization of enteric bacterial pathogens by modulating flagellar motility and nitrate respiration

Tsuyoshi Miki, Shin Kurihara, Takeshi Uemura, Yuta Ami, Masahiro Ito, Takeshi Haneda, Takemitsu Furuchi, Nobuhiko Okada, Tohru Minamino, Yun-Gi Kim

List of Supplemental Material

Supplemental Tables

Supplemental References

Supplemental Figure Legends

Supplemental Figures

Supplemental Tables

Table S1 Genes with decreased expression in Δ*speABCEDF* Δ*potAB* Δ*potFGHI* mutant compared to Δ*speABCEDF*

Function and gene name	Product definition	Fold change
Spermidine/putrescine transport		
potA	spermidine/putrescine transport ATP-binding protein	-12697.70
potB	spermidine/putrescine transport system permease protein	-5750.47
potF	putrescine-binding periplasmic protein precursor	-357.07
potI	putrescine transport system permease protein	-317.45
potG	putrescine transport ATP-binding protein	-294.18
potH	putrescine transport system permease protein	-254.41
potE	putrescine-ornithine antiporter	-3.69
Amino acid metabolism		
tdcD	propionate kinase	-7.51
tdcC	threonine/serine transporter	-5.01
tdcB	threonine dehydratase	-3.76
Carbohydrate metabolism		
malG	maltose transport innner membrane protein	-6.50
malF	maltose transport innner membrane protein	-6.37
malE	periplasmic maltose-binding protein	-5.28
malK	maltose/maltodextrin transport ATP-binding protein	-5.12
lamB	maltoporin	-4.49
malM	maltose operon periplasmic protein	-4.11
malP	maltodextrin phoshorylase	-3.59

malQ	4-alpha-glucanotransferase	-3.19
frdB	fumarate reductase, iron-sulfur protein	-2.43
frdD	fumarate reductase subunit D	-2.37
malZ	alpha-glucosidase	-2.14
Nitrate respiration		
nirC	nitrite transporter	-4.39
narJ	respiratory nitrate reductase 1 delta chain	-4.02
napC	cytochrome c-type protein	-3.03
narH	respiratory nitrate reductase 1 beta chain	-2.97
napG	ferredoxin-type protein	-2.71
napH	ferredoxin-type protein	-2.42
nirB	nitrite reductase large subunit	-2.12
nirD	nitrite reductase (NAD(P)H) small subunit	-2.12
Flagellar motility		
fliO	flagellar T3SS protein	-4.15
fliP	flagellar T3SS protein	-3.83
flhA	flagellar T3SS protein	-3.66
fliI	flagellar T3SS ATP synthase	-3.36
fliH	flagellar T3SS protein	-3.30
fliG	flagellar C-ring protein	-3.28
fliK	flagellar hook-length control protein	-3.24
fliM	flagellar C-ring protein	-3.19
fliN	flagellar C-ring protein	-3.17
fliF	flagellar MS-ring protein	-3.03
fliL	flagellar stator associate protein	-2.96
flgH	flagellar L-ring protein	-2.82
flhB	flagellar T3SS protein	-2.81
flgF	flagellar rod protein	-2.70

flgI	flagellar P-ring protein	-2.65
flgJ	flagellar biosynthesis protein/ flagellar muramidase	-2.62
flgE	flagellar hook protein	-2.51
fliJ	flagellar T3SS protein	-2.49
fliA	flagellum-specific sigma 28 factor	-2.41
flgC	flagellar rod protein	-2.35
flgK	hook-filament junction protein/ HAP1	-2.29
flgA	flagellar periplasmic chaperone	-2.29
fliQ	flagellar T3SS protein	-2.28
flgG	flagellar rod protein	-2.24
flgB	flagellar rod protein	-2.23
fliZ	positive regulator for flagellar gene expression	-2.16
fliR	flagellar T3SS protein	-2.15
fliS	flagellar export chaperone specific for flagellin	-2.10
flgD	flagellar hook cap protein	-2.10
flgL	flagellar hook-filament junction protein/ HAP3	-2.09
Sulfur metabolism		
dmsB	anaerobic dimethyl sulfoxide reductase chain A precursor	-3.61
Chemotaxis		
mcpB	methyl-accepting chemotaxis protein	-3.57
tsr	methyl-accepting chemotaxis protein I, serine sensor receptor	-3.24
cheR	chemotaxis protein methyltransferase	-2.87
trg	methyl-accepting chemotaxis protein III	-2.74
cheA	chemotaxis protein	-2.57
motB	motility protein B	-2.34

cheB	chemotaxis response regulator protein-glutamate methylesterase	-2.24
tcp	methyl-accepting chemotaxis protein	-2.22
cheM	methyl-accepting chemotaxis protein II	-2.22
Nucleotide metabolism		
nrdD	anaerobic ribonucleoside-triphosphate reductase	-3.55
uraA	uracil permease	-2.54
Transcription		
rtsA	regulator of SPI-1	-2.88
Peptide degradation		
pepT	tripeptide aminopeptidase	-2.54
Porphyrin and chlorphyll metabolism		
cysG	siroheme synthase	-2.25
Peptidoglycan biosynthesis and degradation		
dniR	membrane-bound lytic murein transglycosylase D	-2.21
Nitrotoluene degradation		
hypO	hydrogenese-2 small chain protein	-2.01
Unknown		
SL1344_0681	uncharacterized protein	-5.98
SL1344_2731	uncharacterized protein	-4.64
SL1344_1194	peptide/nickel transport system ATP-binding protein	-3.58
SL1344_2713	uncharacterized protein	-3.08
yghW	uncharacterized protein	-2.75

SL1344_1192	peptide/nickel transport system permease protein	-2.74
SL1344_1195	peptide/nickel transport system ATP-binding protein	-2.73
yhbU	hypothetical protease	-2.70
yhjH	uncharacterized protein	-2.63
hybA	hydrogenase-2 small subunit	-2.47
ycgR	uncharacterized protein	-2.41
yjjW	uncharacterized protein	-2.39
srfA	virulence protein	-2.38
SL1344_2751	uncharacterized protein	-2.31
SL1344_1981	putative DNA-binding protein	-2.30
ybjO	uncharacterized protein	-2.25
SL1344_2730	hypothetical ATPase	-2.25
SL1344_3747	hypothetical sugar (pentulose and hexulose) kinase	-2.23
SL1344_2714	uncharacterized protein	-2.03
yhjG	AsmA family protein	-2.01

Table S2 Genes with increased expression in Δ*speABCEDF* Δ*potAB* Δ*potFGHI* mutant compared to Δ*speABCEDF*

Function and gene name	Product definition	Fold change
Amino acid metabolism		
ilvM	acetolactate synthase II small subunit	6.98
leuD	3-isopropylmalate dehydratase small subunit	6.17
hisC	histidinol-phosphate aminotransferase	5.14
hisI	phosphoribosyl-AMP cyclohydrolase/phophoribosyl-ATP pyrophosphohydrolase	5.05
leuC	3-isopropylmalate dehydratase large subunit	5.03
hisH	imidazole glycerol-phosphate synthase subunit	4.71

hisB	imidazoleglycerol-phosphate dehydratase/histidinol-phosphatase	4.54
lctP	L-lactate permease	4.38
hisG	ATP phophoribosyltransferase	4.36
leuB	3-isopropylmalate dehydrogenase	4.24
hisA	phosphoribosylformimino-5-aminoimidazole carboxamide ribotide isomerase	4.22
pheA	chorismate mutase/prephenate dehydratase	4.19
hisF	imidazole glycerol-phosphate synthase subunit	4.09
ilvD	dihydroxyacid dehydratase	3.98
ilvG	acetolactate synthase large subunit	3.86
hisD	histidinol dehydrogenase	3.62
thrA	bifunctional aspartokinase/homoserine dehydrogenase 1	3.58
leuA	2-isopropylmalate synthase	3.30
ilvA	threonine deaminase	3.09
ilvE	branched-chain amino acid aminotransferase	3.09
artJ	arginine transport system substrate-binding protein	2.76
thrC	threonine synthase	2.52
sfbA	D-methionine transport system substrate-binding protein	2.45
ilvN	acetolactate synthase small subunit	2.36
thrB	homoserine kinase	2.30
argC	N-acetyl-gamma-glutamyl-phosphate reductase	2.22
ilvC	ketol-acid reductoisomerase	2.16
argG	argininosuccinate synthetase	2.06
dadA	D-amino-acid dehydrogenase	2.06
Infection		
ssaI	type III secretion system apparatus	5.39
Conjugal transfer		

traE	conjugal transfer protein	4.59
traF	conjugal transfer protein	2.91
Signal transduction		
uspA	universal stress protein A	3.12
phoH	phosphate starvation-inducible protein	2.38
Unclassified metabolism		
ahpC	NADH-dependent peroxiredoxin subunit C	2.95
Carbohydrate metabolism		
gltA	citrate synthase	2.74
icdA	isocitrate dehydrogenase	2.17
Transcription		
crl	sigma factor-binding protein	2.68
dksA	DnaK suppressor protein	2.58
Toxin-antitoxin system		
bssS	biofilm regulator	2.35
Replication and repair		
parA	plasmid partition protein A	2.12
Unknown		
ybfA	uncharacterized protein	7.26
yecG	uncharacterized protein	5.14
yeeI	MtfA peptidase	3.99
ydeW	hypothetical regulatory protein	3.82
nlpC	hypothetical lipoprotein	3.20
SL1344_2626	uncharacterized protein	2.80

yahO	uncharacterized protein	2.65
yqgB	uncharacterized protein	2.53
SL1344_1197	uncharacterized protein	2.50
SL1344_2583	uncharacterized protein	2.40
SL1344_0713	uncharacterized protein	2.36
osmY	uncharacterized protein	2.34
SL1344_0350	uncharacterized protein	2.34
ybjN	uncharacterized protein	2.31
gltL	glutamate/aspartate transport ATP-binding protein	2.29
SL1344_2584	DNA-damage-inducible protein I	2.26
SL1344_3334	uncharacterized protein	2.25
yeaC	uncharacterized protein	2.23
cib	colicin Ib protein	2.22
yeaA	peptide-methionine (R)-S-oxide reductase	2.16
ybdD	uncharacterized protein	2.15
yehE	uncharacterized protein	2.14
yafD	uncharacterized protein	2.13
SL1344_1470	hypothetical regulatory protein	2.13
cybC	soluble cytochrome b562	2.11
SL1344_3141	uncharacterized protein	2.09
SL1344_1635	uncharacterized protein	2.02

Table S3 Bacterial strains and plasmids used in this study

**Strain or plasmid**	**Genotype**	**Reference**
**Strain**		
***Salmonella enterica* serovar Typhimurium (*S*Tm)**		
SL1344	Wild-type *S*Tm, *hisG*	(1)
T643	SL1344 Δ*speABCEDF*::*kan*	(2)

	SL1344 Δ*speABCEDF* Δ*potAB*	
T799	Δ*potFGHI*::*cat*	(2)
S156	SL1344 *napF*::*lacZ*	This study
S157	T799 *napF*::*lacZ*	This study
S386	S157 harboring pMW118	This study
S387	S157 harboring pMW-*potAB*	This study
S388	S157 harboring pMW-*potFGHI*	This study
S349	SL1344 Δ*narP*::*kan*	This study
S358	S349 *napF*::*lacZ*	This study
S364	S349 *napF*::*lacZ* harboring pMW-*narP*	This study
S424	SL1344 *narP*::*lacZ*	This study
S425	T799 *narP*::*lacZ*	This study
S479	S156 harboring pBAD-*narP*	This study
S480	S157 harboring pBAD-*narP*	This study
S481	S358 harboring pBAD-*narP*	This study
S490	SL1344 harboring pBAD-*narP*	This study
S491	T799 harboring pBAD-*narP*	This study
S223	SL1344 *fliC*::*lacZ*	This study
TM1222	SL1344 Δ*fliA*::*cat*	This study
S240	TM1222 *fliC*::*lacZ*	This study
S724	T799 *fliC*::*lacZ*	This study
S725	S724 harboring pMW118	This study
S726	S724 harboring pMW-*potAB*	This study
S727	S724 harboring pMW-*potFGHI*	This study
T523	SL1344 Δ*fliGHI*::*cat*	(3)
TM1172	SL1344 Δ*fliC*::*kan*	(3)
T330	SL1344 harboring pACYC-gfp	(4)
T805	SL1344 Δ*speABCEDF* Δ*potAB* Δ*potFGHI*	(2)
S786	T805 harboring pACYC-gfp	This study
T273gfp	T273 harboring pACYC-gfp	This study

T984	SL1344 Δ*narKGHJI* Δ*napFDAGHBC*::*cat*	This study
S112	SL1344 Δ*narKGHJI* Δ*napFDAGHBC*	This study
S412	SL1344 Δ*speABCEDF* Δ*potAB* Δ*potFGHI* Δ*narKGHJI*::*cat* Δ*napFDAGHBC*	This study
T273	Δ*fliGHI*	(3)
S399	SL1344 Δ*speABCEDF* Δ*potAB* Δ*potFGHI* Δ*fliGHI*::*cat*	This study
S698	SL1344 Δ*narKGHJI* Δ*napFDAGHBC* Δ*fliGHI*::*cat*	This study
S603	SL1344 Δ*narKGHJI* Δ*napFDAGHBC* Δ*fliGHI*::*cat* Δ*speABCEDF* Δ*potAB* Δ*potFGHI*	This study
T249	SL1344 Δ*invG* Δ*ssaV*::*cat*	(5)
S395	SL1344 Δ*invG* Δ*ssaV*::*cat* Δ*speABCEDF* Δ*potAB* Δ*potFGHI*	This study
S788	SL1344 Δ*invG* Δ*ssaV*::*cat* Δ*narKGHJI* Δ*napFDAGHBC* Δ*fliGHI* Δ*speABCEDF* Δ*potAB* Δ*potFGHI*	This study
**Adherent-invasive *Escherichia coli* (AIEC)**		
LF82	*E. coli* isolate from an ileal biopsy sample of a Crohn's disease patient	(6)
**Plasmid**		
pMW118	Low-copy-number expression vector	Nippon Gene
pMW-*potAB*	pMW118 containing *potAB*, expressing PotAB	(2)
pMW-*potFGHI*	pMW118 containing *potAB*, expressing PotAB	(2)

pMW-*narP*	pMW118 containing *narP*, expressing NarP	This study
pLD-*lacZ*Ω	Integration plasmid with a promoterless *lacZ* gene	(7)
pLD-*napFZ*	*napF*::*lacZ* transcriptional fusion in pLD-*lacZΩ*	This study
pLD-*narPZ*	*narP*::*lacZ* transcriptional fusion in pLD-*lacZΩ*	This study
pLD-*fliCZ*	*fliC*::*lacZ* transcriptional fusion in pLD-*lacZΩ*	This study
pBAD/His A	N-terminal 6xHis-tagged protein-expressing plasmid	Invitrogen
pBAD-*narP*	pBAD/His A encoding *narP*	This study

Table S4 Oligonucleotide primers used in this study

Name	Sequence (5' to 3')
qfliL-FW	GATTTGGATACCTTTGCTGGTGTT
qfliL-RV	TAGCGTTCGTGGTCGGTTG
qflgB-FW	TTCAGCAGGAAGCGCTAAATC
qflgB-RV	GTCCACGCACCATCACCTT
qfliA-FW	GGCGTAATGCGACGGAAA
qfliA-RV	AGAGAAAAGTTGGCTGTTGTTGG
qSL lamB-F	GGTATTGGCTGGACGGGAAG
qSL lamB-R	CCAGTTTCAGTTCCGCATAGGT
qSL malE-F	TTGAAGCCCTCTCCCTGATTT
qSL malE-R	GCCGACTTCCCTTTCACTTTT
qSL malP-F	CTGGCGGATTACGAGGTCA
qSL malP-R	TGTGGTTGGTGTAGGCGAAG

qtsr-FW	GGTCATTATCGCCGTCTGGT
qtsr-RV	ACTGCCCCATTTCGTTGG
qmotA-FW	CCATCAAAGGCACGATGAAA
qmotA-RV	AGCAACGCCAGCAAATCC
qcheA-FW	GATGGCGGTGAATGAAAACA
qcheA-RV	GACCTGTTCCGCAGTTGAGA
qcheB-FW	CGCAGCTTGGCATTCGT
qcheB-RV	GCCGCCATCGGTTTATGT
qSL narJ-F	TGGGAAGAAGAGCAGGTGAAA
qSL narJ-R	GCAAAACGACGCTGGTGAG
qSL napB-F	GGTGGTAAGTGGTGCGGTTT
qSL napB-R	CGGCATACGTTCCTGCTCTT
qSL nirC-F	CCATCAGCCACGGTCAAA
qSL nirC-R	GTAAAGCAGGGCGACAAACAC
rpoD-20-F	ACATGGGTATTCAGGTAATGGAAGA
rpoD-20-R	CRGTGCTGGTGGTATTTTCA
SL Pro-napF-SalI	AAAGTCGACAGTATTTCCTTTCTGCTG
SL Rev-napF-BamHI	AAAGGATCCAAATAGGTCGTTTCTCTG
SL narP-red-FW	GAAAACAATAATCATTCTAATAAAACTCAGGAGATACAGCGTGTAGGCTGGAGCTGCTTC
SL narP-red-RV	CCTGACGGCCCGGTGGCGCGGCGCCACCGGGCCTGGCTTGCATATGAATATCCTCCTTAG
SL narP-KpnI-FW	AAAGGTACCCGTTCAGCTAATTACTCG
SL narP-SalI-RV	AAAGTCGACGAAATAAGACCCTGACGG

SL Pro-narP-SalI	AAAGTCGACTGCCAGAATGTTTACTGC
SL Rev-narP-BamHI	AAAGGATCCCACCTGAAAAGGTGTTAC
SL narP-XhoI-FW	AAACTCGAGCCTGAAGTAACACCTTTTC
SL narP-EcoRI-RV	AAAGAATTCTTACTGCATTCCGCGTGT
PronapF-F	CGGCAACAGCATTTACCTCTATC
PronapF-R	CTTCCATAACGACACGCTTCC
SL narK-red-FW	CCTAATGTGGCAGACATCAAATCAAGAATCAGAGGTGTCTGTG TAGGCTGGAGCTGCTTC
SL narI-red-RV	AAAACTCCGCCGAAGCGGAGTTTAGGGCATCGAGAGAAAACAT ATGAATATCCTCCTTAG
SL napF-red-FW	CACTCCCTATGGAAGCGTGTCGTTATGGAAGGGCGATATCGTGT AGGCTGGAGCTGCTTC
SL napC-red-RV	AGATCTCTGGCTTCAAGCATTCCCACTCATCAACGTCCTGCATA TGAATATCCTCCTTAG
fliA-red-FW	CTGTATACCGCTGAAGGTGTAATGGATAAACACTCGCTGTGTGT AGGCTGGAGCTGCTTC
fliA-red-RV	TACCCAGTTTGGTGCGTAATCGTTTGATGGCCTGACTATGCATA TGAATATCCTCCTTAG
fliC-Pro-SalI	AAAGTCGACTTTTTGTCGCCAGCGACTGTG
fliC-Rev-BamHI	AAAGGATCCTCTGGGTCAACAGCGACAGGC

Supplemental References

1. Hoiseth SK, Stocker BA. 1981. Aromatic-dependent Salmonella typhimurium are

non-virulent and effective as live vaccines. Nature 291:238-9.

2. Miki T, Uemura T, Kinoshita M, Ami Y, Ito M, Okada N, Furuchi T, Kurihara S, Haneda T, Minamino T, Kim YG. 2024. Salmonella Typhimurium exploits host polyamines for assembly of the type 3 secretion machinery. PLoS Biol 22:e3002731.
3. Nakamura N, Hoshino Y, Shiga T, Haneda T, Okada N, Miki T. 2020. A Peptidoglycan Amidase Activator Impacts Salmonella enterica Serovar Typhimurium Gut Infection. Infect Immun 88.
4. Fujimoto M, Goto R, Hirota R, Ito M, Haneda T, Okada N, Miki T. 2018. Tat-exported peptidoglycan amidase-dependent cell division contributes to Salmonella Typhimurium fitness in the inflamed gut. PLoS Pathog 14:e1007391.
5. Fujimoto M, Goto R, Haneda T, Okada N, Miki T. 2018. Salmonella enterica Serovar Typhimurium CpxRA Two-Component System Contributes to Gut Colonization in Salmonella-Induced Colitis. Infect Immun 86.
6. Darfeuille-Michaud A, Neut C, Barnich N, Lederman E, Di Martino P, Desreumaux P, Gambiez L, Joly B, Cortot A, Colombel JF. 1998. Presence of adherent Escherichia coli strains in ileal mucosa of patients with Crohn's disease. Gastroenterology 115:1405-13.
7. Miki T, Okada N, Danbara H. 2004. Two periplasmic disulfide oxidoreductases, DsbA and SrgA, target outer membrane protein SpiA, a component of the Salmonella pathogenicity island 2 type III secretion system. J Biol Chem 279:34631-42.

Supplemental Figure Legends

Figure S1. Intracellular contents of polyamines of *S*Tm grown in LB medium.

Measurement of intracellular contents of putrescine, spermidine, and cadaverine from *S*Tm WT, Δ*speABCEDF*, and Δ*speABCEDF* Δ*potAB* Δ*potFGHI* grown in LB medium until logarithmic growth phase. ND, not detected. Bars, median. A one-way ANOVA followed by Dunnett's multiple comparisons test. $P > 0.05$ not significant (ns), $P < 0.05$ (*), $P < 0.01$ (**), $P < 0.001$ (***), $P < 0.0001$ (****).

Figure S2. Comparative expression of *narKGHJI* and *napFDAGHBC* operon between the Δ*speABCEDF* and Δ*speABCEDF* Δ*potAB* Δ*potFGHI.*

Heatmap of DEGs for *narKGHJI* and *napFDAGHBC* operons. Genes involved in nitrate reductase were selected and displayed as a heatmap. Use the transcripts per million (TPM) values of selected genes in RNA-seq samples to draw a heatmap. The adjusted *P* values are also visualized as a bar plot.

Figure S3. RT-qPCR analysis of *napB* and *narJ.*

(A and B) Transcripts of the *napB* (A) and *narJ* (B) gene are shown as relative amounts to Δ*speABCEDF* Δ*potAB* Δ*potFGHI* in *S*Tm grown in M9 medium supplemented with putrescine or spermidine at 3 mM. Bars represent mean ± SD from at least three independent experiments. A one-way ANOVA followed by Dunnett's multiple comparisons test. $P > 0.05$ not significant (ns), $P < 0.05$ (*), $P < 0.01$ (**), $P < 0.001$ (***), $P < 0.0001$ (****).

Figure S4. Comparative expression of genes involved in flagellar motility and chemotaxis between the Δ*speABCEDF* and Δ*speABCEDF* Δ*potAB* Δ*potFGHI.*

Heatmap of DEGs for flagellar regulon genes involved in motility and chemotaxis. Genes involved in flagellar motility and chemotaxis were selected and displayed as a heatmap. Use the transcripts per million (TPM) values of selected genes in RNA-seq samples to draw a heatmap. The adjusted *P* values are also visualized as a bar plot.

Figure S5. *S*Tm Δ*narKGHIJI* Δ*napFDAGHBC* mutants are outcompeted by wild-type strains in the gut colonization.

C57BL/6 SPF mice were pre-treated with 25 mg of streptomycin by oral gavage 24 h before

oral infection with *S*Tm (1:1 mixture of strain 1 and strain 2). Feces were collected on day 4 post infection. The CI of *S*Tm loads recovered from feces and cecal content as determined by selective plating. Bars, median. Wilcoxon signed-rank test to compare the median of CI values with a hypothetical median of 1, which indicates that both strains colonize at equivalent levels. $P > 0.05$ not significant (ns), $P < 0.05$ (*), $P < 0.01$ (**), $P < 0.001$ (***), $P < 0.0001$ (****).

Figure S6. Two T3SSs are required for sustained gut colonization.

(A) Experimental scheme of *S*Tm oral infection in the streptomycin-treated mouse model. C57BL/6 SPF mice were pre-treated with 25 mg of streptomycin by oral gavage 24 h before oral infection with *S*Tm. Feces were collected on day 1 postinfection (1 dpi). Mice were euthanized on day 4 post infection (4 dpi), and feces were collected. (B) Bacterial loads recovered from feces as determined by selective plating. Bars, median. Two-tailed Mann Whitney-U tests to compare two groups in each panel. $P > 0.05$ not significant (ns), $P < 0.05$ (*), $P < 0.01$ (**), $P < 0.001$ (***), $P < 0.0001$ (****).

Figure S7. Luminal spermidine improves the gut colonization of *S*Tm strain while maintaining polyamine homeostasis.

(A) Experimental scheme of *S*Tm oral infection in the streptomycin-treated dysbiotic mouse model. Two percent of DSS-drinking C57BL/6 SPF mice were pre-treated with 25 mg of streptomycin by oral gavage 24 h before oral infection with *S*Tm Δ*invG* Δ*ssaV* mutant (an infectious dose of 5×10^7). Mice were treated with 1% spermidine (SPD) in the drinking water and euthanized on day 4 post-infection (4 dpi), and feces were collected. (B) Bacterial loads recovered from feces was determined by selective plating. Bars, median. Two-tailed Mann–Whitney U tests to compare two groups in each panel. $P > 0.05$ not significant (ns), P

< 0.05 (*), $P < 0.01$ (**), $P < 0.001$ (***), $P < 0.0001$ (****).

Figure S8. Microbiota-derived and inflammation-induced intestinal luminal polyamines support gastrointestinal infection with enteric bacterial pathogens, including *S*Tm, by modulating activities of flagellar motility and nitrate respiration. In the gut lumen, the infected *S*Tm imports microbiota-derived polyamines, which are required for the expression of genes involved in flagellar motility and nitrate respiration. In addition, gut inflammation induces the luminal levels of polyamines. Thus, *S*Tm persistently colonizes the gut lumen through bacterial expansion. Activation of flagellar motility also contributes to the invasion of intestinal epithelial cells (IECs) and the induction of inflammatory responses. Furthermore, the uptake of polyamines is required for the assembly of the T3SS needle, thereby accelerating *S*Tm gut infection (2). In conclusion, gastrointestinal infection with enteric bacterial pathogens may be strongly influenced by intestinal luminal levels of polyamines. Created with BioRender.com (Agreement number: XP28B3HUI5).

Figure S1

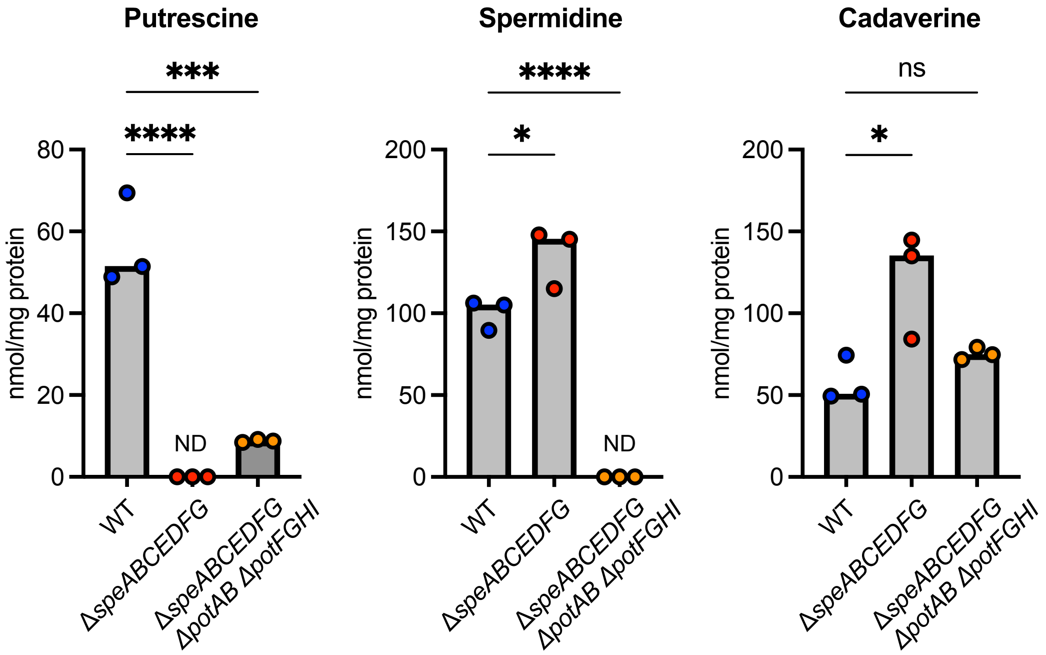

Putrescine
Spermidine
Cadaverine
***
****
*
ns
ND
nmol/mg protein
0
20
40
60
80
50
100
150
200
WT
ΔspeABCEDFG
ΔspeABCEDFG ΔpotAB ΔpotFGHI

Figure S2

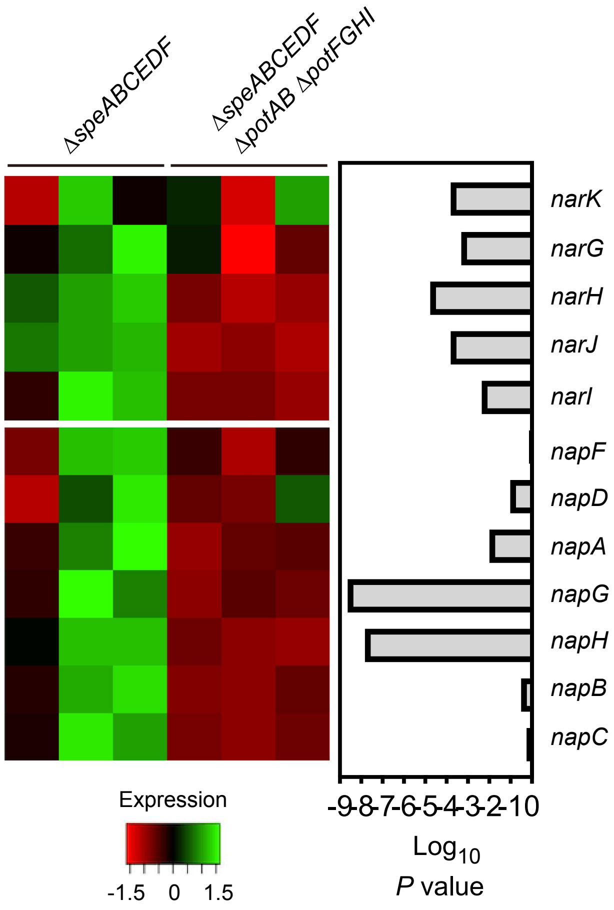

ΔspeABCEDF
ΔspeABCEDF
ΔpotAB ΔpotFGHI
narK
narG
narH
narJ
narI
napF
napD
napA
napG
napH
napB
napC
-9-8-7-6-5-4-3-2-10
Log10
P value
Expression
-1.5 0 1.5

Figure S3

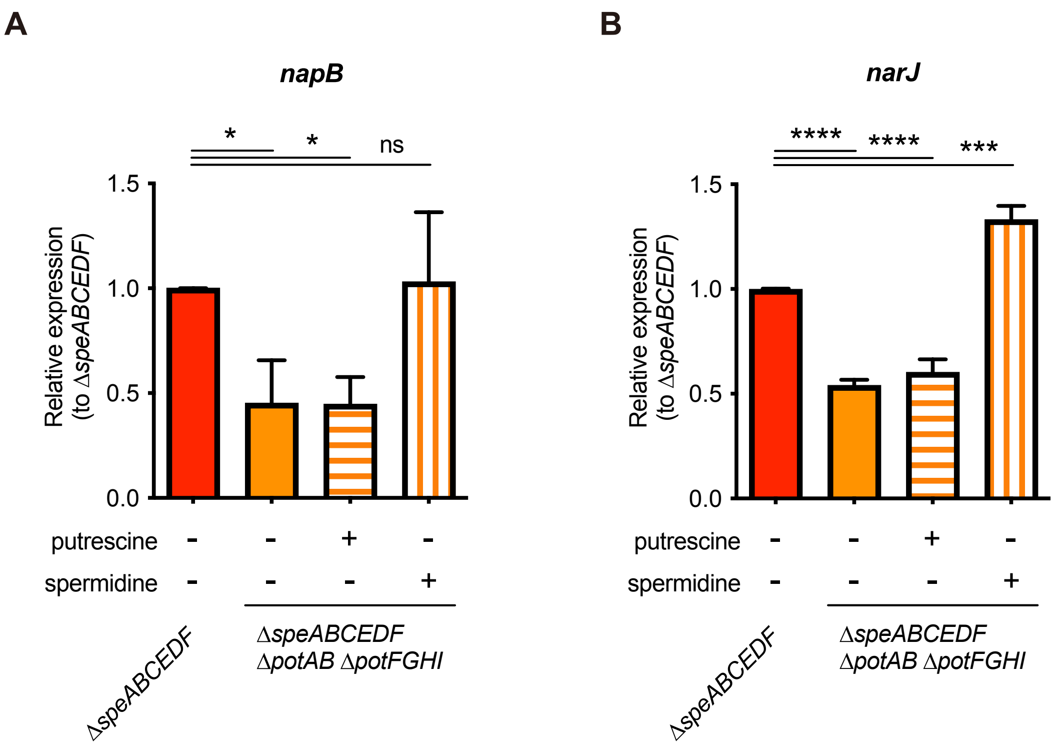
A
napB
*
*
ns
1.5
1.0
0.5
0.0
Relative expression
(to ΔspeABCEDF)
putrescine - - + -
spermidine - - - +
ΔspeABCEDF
ΔspeABCEDF
ΔpotAB ΔpotFGHI
B
narJ
****
****
***
1.5
1.0
0.5
0.0
Relative expression
(to ΔspeABCEDF)
putrescine - - + -
spermidine - - - +
ΔspeABCEDF
ΔspeABCEDF
ΔpotAB ΔpotFGHI

Figure S4

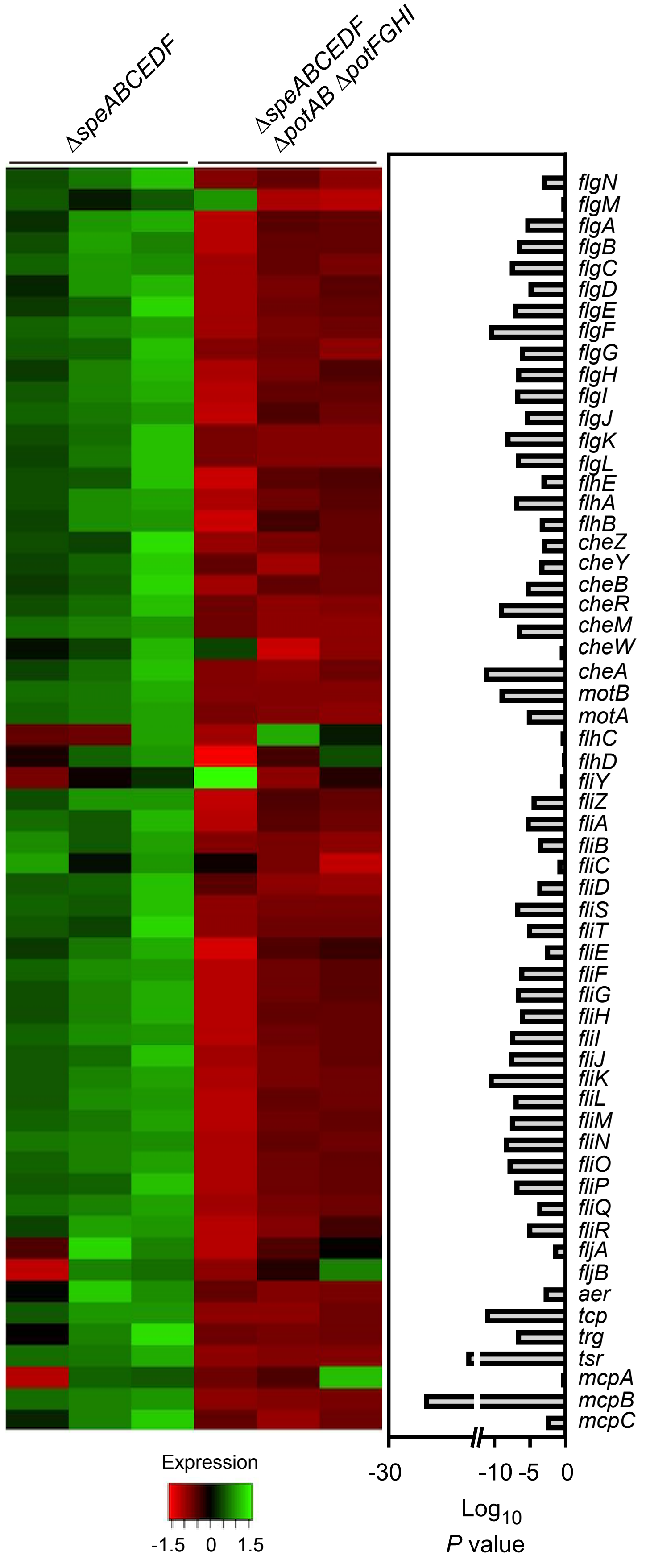
ΔspeABCEDF
ΔspeABCEDF ΔpotAB ΔpotFGHI
flgN
flgM
flgA
flgB
flgC
flgD
flgE
flgF
flgG
flgH
flgI
flgJ
flgK
flgL
flhE
flhA
flhB
cheZ
cheY
cheB
cheR
cheM
cheW
cheA
motB
motA
flhC
flhD
fliY
fliZ
fliA
fliB
fliC
fliD
fliS
fliT
fliE
fliF
fliG
fliH
fliI
fliJ
fliK
fliL
fliM
fliN
fliO
fliP
fliQ
fliR
fljA
fljB
aer
tcp
trg
tsr
mcpA
mcpB
mcpC
Expression
-1.5
0
1.5
-30
-10
-5
0
Log10
P value

Figure S5

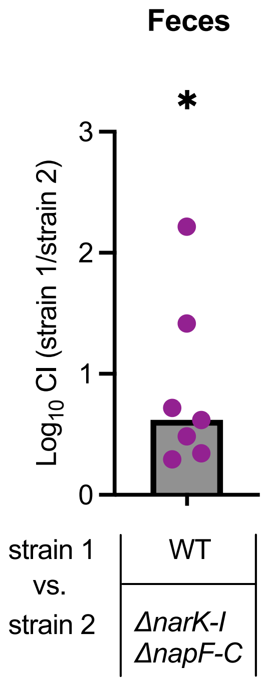

ΔnarK-I: *ΔnarKGHJI*
ΔnapF-C: *ΔnapFDAGHBC*

Figure S6

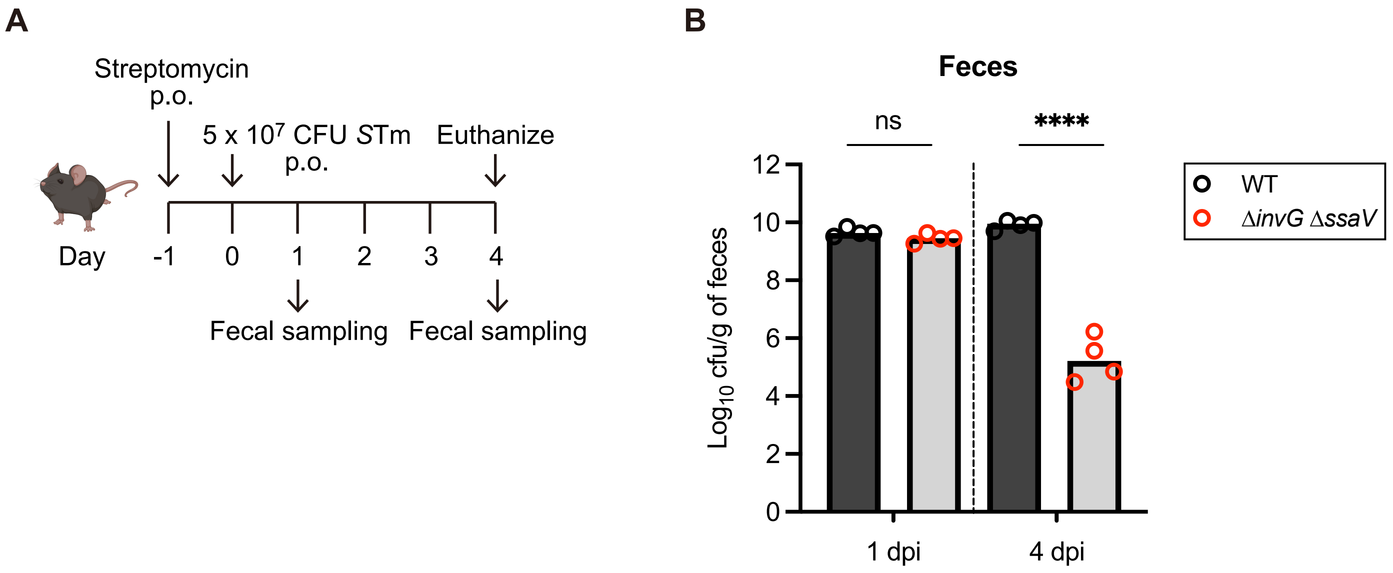
A
Streptomycin
p.o.
5 x 10^7 CFU STm
p.o.
Euthanize
Day
-1
0
1
2
3
4
Fecal sampling
Fecal sampling
B
Feces
ns
****
Log10 cfu/g of feces
0
2
4
6
8
10
12
1 dpi
4 dpi
WT
ΔinvG ΔssaV

Figure S7

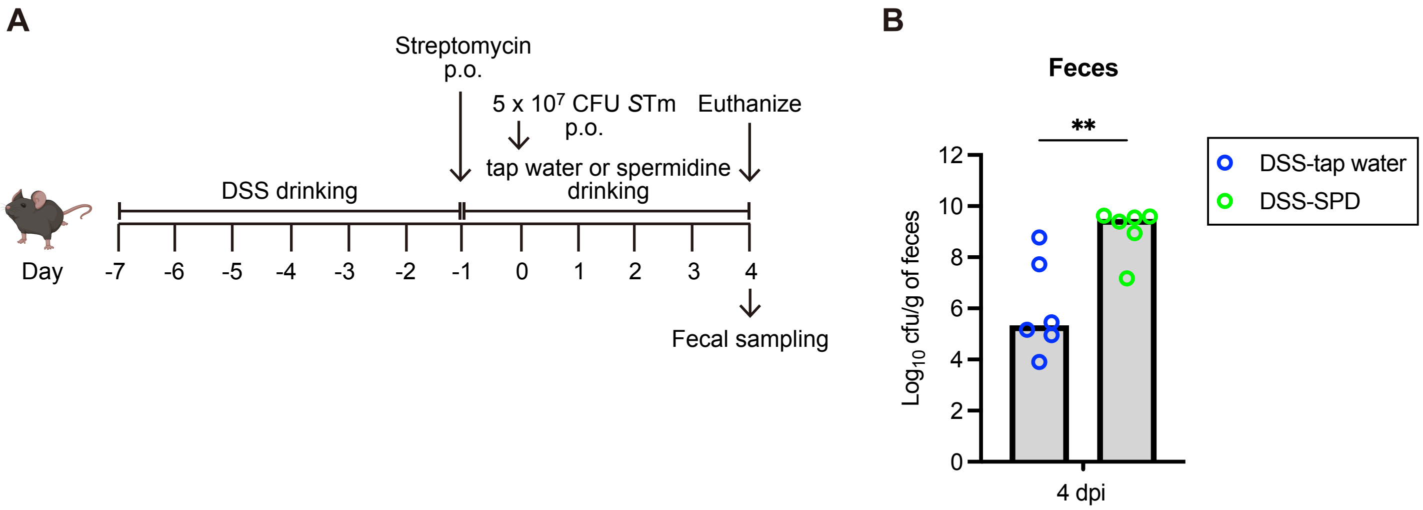
A
Streptomycin
p.o.
5 x 10⁷ CFU STm
p.o.
Euthanize
tap water or spermidine
drinking
DSS drinking
Day
-7
-6
-5
-4
-3
-2
-1
0
1
2
3
4
Fecal sampling
B
Feces
**
12
10
8
6
4
2
0
Log10 cfu/g of feces
4 dpi
DSS-tap water
DSS-SPD

Figure S8

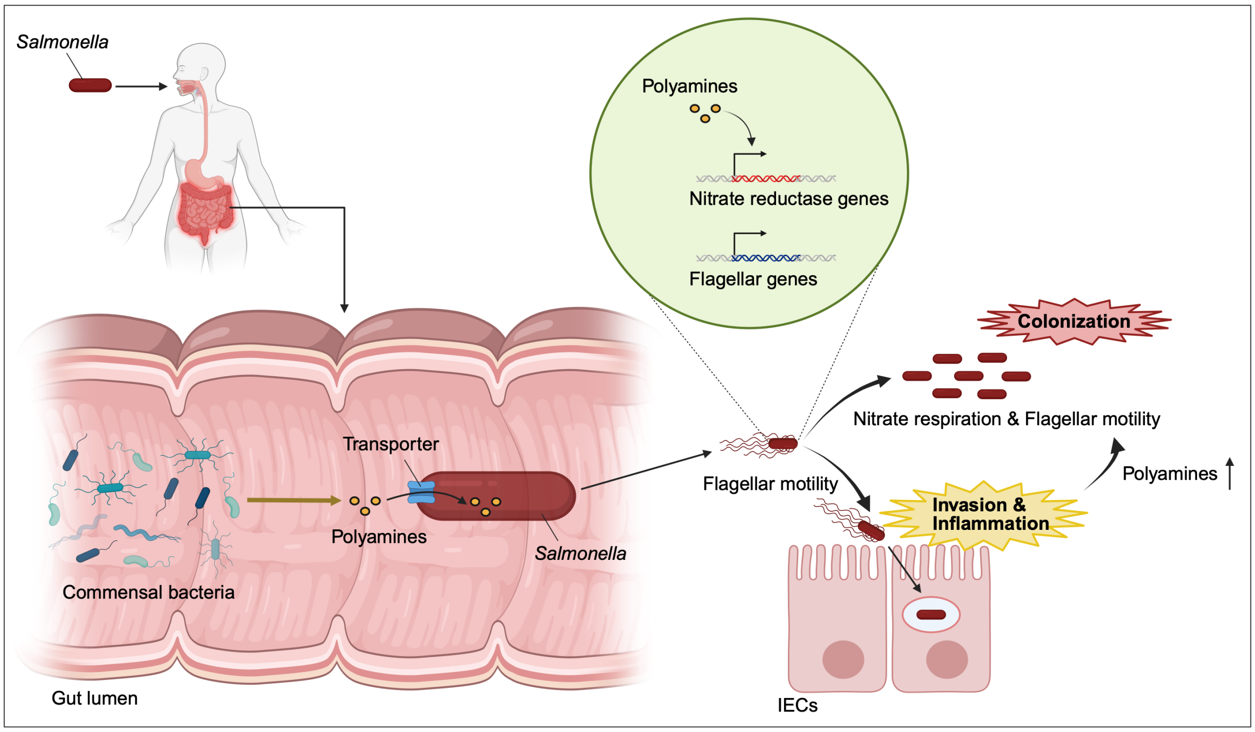

Salmonella
Polyamines
Nitrate reductase genes
Flagellar genes
Colonization
Nitrate respiration & Flagellar motility
Transporter
Flagellar motility
Polyamines
Invasion & Inflammation
Polyamines
Salmonella
Commensal bacteria
Gut lumen
IECs
